# Comparative susceptibility of SARS-CoV-2, SARS-CoV, and MERS-CoV across mammals

**DOI:** 10.1038/s41396-023-01368-2

**Published:** 2023-01-23

**Authors:** Meng Li, Juan Du, Weiqiang Liu, Zihao Li, Fei Lv, Chunyan Hu, Yichen Dai, Xiaoxiao Zhang, Zhan Zhang, Gaoming Liu, Qi Pan, Yang Yu, Xiao Wang, Pingfen Zhu, Xu Tan, Paul A. Garber, Xuming Zhou

**Affiliations:** 1 Key Laboratory of Animal Ecology and Conservation Biology, Institute of Zoology, Chinese Academy of Sciences, Beijing, 100101 China; 2 University of Chinese Academy of Sciences, Beijing, 100049 China; 3 School of Life Sciences, University of Science and Technology of China, Anhui, China; 4 Beijing Advanced Center for Structural Biology, Beijing Frontier Innovation Center, School of Pharmaceutical Sciences, Tsinghua-Peking Center for Life Sciences, Tsinghua University, 100084 Beijing, China; 5 Department of Anthropology, Program in Ecology, Evolution, and Conservation Biology, University of Illinois, Urbana, IL USA

**Keywords:** Cellular microbiology, SARS-CoV-2

## Abstract

Exploring wild reservoirs of pathogenic viruses is critical for their long-term control and for predicting future pandemic scenarios. Here, a comparative in vitro infection analysis was first performed on 83 cell cultures derived from 55 mammalian species using pseudotyped viruses bearing S proteins from SARS-CoV-2, SARS-CoV, and MERS-CoV. Cell cultures from Thomas’s horseshoe bats, king horseshoe bats, green monkeys, and ferrets were found to be highly susceptible to SARS-CoV-2, SARS-CoV, and MERS-CoV pseudotyped viruses. Moreover, five variants (del69-70, D80Y, S98F, T572I, and Q675H), that beside spike receptor-binding domain can significantly alter the host tropism of SARS-CoV-2. An examination of phylogenetic signals of transduction rates revealed that closely related taxa generally have similar susceptibility to MERS-CoV but not to SARS-CoV and SARS-CoV-2 pseudotyped viruses. Additionally, we discovered that the expression of 95 genes, e.g., *PZDK1* and *APOBEC3*, were commonly associated with the transduction rates of SARS-CoV, MERS-CoV, and SARS-CoV-2 pseudotyped viruses. This study provides basic documentation of the susceptibility, variants, and molecules that underlie the cross-species transmission of these coronaviruses.

## Introduction

Severe acute respiratory syndrome coronavirus 2 (SARS-CoV-2) has posed a considerable threat to public health and the global economy, with more than 500 million cases of human infection and over 6.6 million deaths worldwide as of Jan 2023. SARS-CoV-2 was speculated to have originated in bats and then jump to the human population via an intermediate animal host [1–3]. Although viruses similar to SARS-CoV-2, such as BANAL-20-52 derived from the Malayan horseshoe bat (*Rhinolophus malayanus*), RaTG13 derived from the intermediate horseshoe bat (*Rhinolophus affinis*), and Pangolin‐CoV present in Malayan pangolins (*Manis avania*) [4–6] have been identified, at present the exact animal origin of SARS-CoV-2 remains unclear. Two other coronaviruses that caused epidemics prior to COVID-19 are severe acute respiratory syndrome coronavirus (SARS-CoV), which possibly spread from horseshoe bats to humans through infected palm civets, and Middle East respiratory syndrome coronavirus (MERS-CoV), which was possibly a spillover from bats to humans via infected dromedary camels [7–9]. Although the current threat of both SARS-CoV and MERS-CoV is minimal, we must be vigilant about the potential risks of spillback to humans from their natural reservoirs [10, 11].

SARS-CoV-2 is able to infect a broad spectrum of hosts including dogs, mink, ferrets, otters, hamsters, voles, deer, deer mice, bats, small and large felines, and several nonhuman primates [12**–**14]. However, it remains a challenge to compare the susceptibility of these species because standardized measurements across species have not been employed. In this regard, several studies have predicted the SARS-CoV-2 infection probability by analyzing angiotensin-converting enzyme 2 (*ACE2*) orthologs. For example, Damas et al. utilized the *ACE2* sequences of 410 vertebrate species and found that certain endangered taxa (e.g., red-shanked doucs, proboscis monkeys, rhesus macaques, and Antarctic minke whales) were at the highest risk for SARS-CoV-2 infection [15]. At the same time, crystal structure resolution, surface plasmon resonance analyses, and molecular dynamic simulations have been used to evaluate the binding affinity of different ACE2 orthologs to spike proteins [15**–**17]. While these studies provide valuable information about the likely host range of SARS-CoV-2, their predictions require experimental validation. Moreover, the effects of host factors other than ACE2 associated with viral invasion have been underappreciated in these predictions [15, 18, 19].

Animal infection experiments provide the best opportunity to understand the susceptibility, pathogenicity, and transmissibility of pathogens across different taxa [20**–**23]. However, it is impractical to perform in vivo inoculation studies in a wide range of species, particularly in wildlife. Alternatively, an in vitro infection assay of diverse cell lines has the potential to offer critical insights into the infectivity of SARS-CoV-2 [24, 25]. For instance, Chu et al. assessed the replication kinetics and cytopathic effects of 25 cell lines derived from different species. They demonstrated that SARS-CoV-2 can replicate in non-human primate, cat, rabbit, and pig cells [24]. In another study, airway epithelial cells were collected from 12 animal species, and SARS-CoV-2 was found to replicate efficiently in monkey and cat culture models [25]. Aside from the demand for cellular assays to assess the potential host spectrum, it also is urgent to explore how mutations can affect both the infectivity and transmissibility of SARS-CoV-2 to different species. The continuous adaptive evolution of SARS-CoV-2 has resulted in the rapid emergence of novel mutations; however, it remains unknown how many of these mutations have enhanced the susceptibility of animals to SARS-CoV-2.

Here, we first assessed the potential host range of SARS-CoV, MERS-CoV, and SARS-CoV-2 using pseudotyped viruses and cell cultures derived from 55 mammalian species. The ability of site mutations in S proteins to affect the host range of SARS-CoV-2 was determined using site-directed mutagenesis. We then employed a comparative transcriptomics approach to identify gene expressions that were associated with species’ susceptibility to these viruses. This study provides new information about the plausible host tropism of SARS-CoV, MERS-CoV, and SARS-CoV-2. Moreover, we uncovered the expression of host factors that are likely to affect the cross-species transmission of SARS-CoV-2 and prevent the future spillback of these coronaviruses from humans to other species.

## Material and methods

### Cell culture models

In order to improve the reproducibility of this study, whenever possible, we sampled healthy young adult males of each species to reduce the effects of sex, age, immunity, and related factors. To recapitulate cells with the highest susceptibility, we isolated primary cell cultures used in this study (Table S1) from multiple tissues (i.e., kidney, lung, brain, spleen, and heart) of pets, livestock, and wildlife animals based on protocols previously described [26]. Animals were euthanized using CO_2_ or pentobarbital calcium (200 mg/kg). The carcasses were dissected, followed by the collection of kidneys, lungs, hearts, spleens, or brains under aseptic conditions. To avoid drying, all tissues were placed in sterile PBS supplemented with a 2% Penicillin–streptomycin solution (Gibco, USA) until cell extraction. Each tissue was transferred into a 35 mm dish and minced into tiny pieces using dissecting scissors. Tissue fragments were further transferred into a 50 mL conical tube, followed by enzyme digestion using 0.25% EDTA-trypsin (Gibco, USA) at 37 °C for 30 min. During the digestion, the mixture was shaken vigorously every 10 min. Trypsin digestion was stopped by the addition of FBS into the conical tube. The mixture containing tissue fragments was pipetted up and down for 3 min using a 5 mL pipettor to break up the clumps. The resulting solution was spun down at 250 *g* for 5 min at 4 °C. Pellet cells were then collected, resuspended, counted, and seeded into 100 mm Petri-dishes. All dishes were placed at 37 °C with 5% CO_2_. The cells were checked daily for cell growth and contamination. Regular cell passages were performed when the cells reached confluence. All primary cells were isolated and cultured in DMEM-F12 with 10% FBS, a 1% penicillin–streptomycin solution, and 20 mM HEPES. All experiments were conducted in a Biosafety Level 2 (BSL-2) facility and approved by the Animal Ethics Committee of the Institute of Zoology, Chinese Academy of Sciences (permission no: IOZ-IACUC-2021-163).

Cell lines including Huh-7 (*Homo sapiens*, liver), A549 (*Homo sapiens*, lung), SW480 (*Homo sapiens*, colon), HEK-293T (*Homo sapiens*, embryonic kidney), Vero-E6 (*Cercopithecus aethiops*, kidney), Marc-145 (*Cercopithecus aethiops*, kidney), OK (*Monodelphis domestica*, kidney), BHK-21(*Mesocricetus auratus*, kidney), SP2/0 (*Mus musculus*, myeloma cells), NIH3T3 (*Mus musculus*, embryo), MDBK (*Bos taurus*, kidney), PK-15 (*Sus scrofa*, kidney), MDCK (*Canis familiaris*, kidney), F81 (*Felis catus*, kidney), and Mv.1.lu (*Neovison vison*, lung) cells were cultured in DMEM. HT-29 (*Homo sapiens*, colon) cell lines were cultured in RPMI-1640 (Gibco, USA). NEF (Naked-mole rat, embryo) cell lines were cultured in DMEM-F12. Immortalized bat cell lines PaKi (*Pteropus alecto*, kidney), PaLu (*Pteropus alecto*, lung), and PaBr (*Pteropus alecto*, brain) were kindly provided by Dr. Linfa Wang (Duke-NUS Medical School), and cultured in DMEM-F12. All cell lines were incubated at 37 °C in the presence of 5% CO_2_ using specified culture mediums that were supplemented with 10% fetal bovine serum (FBS, Gibco, USA), a 1% penicillin–streptomycin solution, and 20 mM hydroxyethylpiperazine ethane sulfonic acid (HEPES, Gibco, USA).

### Plasmids and site-directed mutagenesis

pVSV-eGFP-dG (Addgene #31842), pCAG-VSVN (Addgene #64087), pCAG-VSVP (Addgene #64088), pCAG-VSVL (Addgene #64085), pCAG-VSVG (Addgene #64084), and pCAG-T7pol (Addgene #59926) plasmids were acquired from the Addgene. Human codon-optimized S genes of SARS-CoV (SARS coronavirus Tor2, GenBank accession No. NC_004718.3), SARS-CoV-2 (SARS coronavirus 2 Wuhan-Hu-1, GenBank accession No. NC_045512.2), and MERS-CoV (MERS coronavirus HCoV-EMC, GenBank accession No. NC_019843.3) were synthesized and cloned into a pcDNA3.1 vector for pseudotyped virus generation. The constructed plasmids were named pcDNA3.1-SARS-S, pcDNA3.1-SARS2-S, and pcDNA3.1-MERS-S, respectively. The expression of S proteins in HEK-293T cells was verified using Western Blot. Site-directed mutagenesis was performed using pcDNA3.1-SARS2-S as a template. Forward primers were designed with *Tm* of ~75 °C and the mutations were centered in the middle, while correspondence reverses complementary sequences were selected as reverse primers. A volume of 20 μL PCR mix was prepared to contain 10 μL 2× Phanta Max Master Mix (Vazyme, PR China), 10 pmol of forward and reverse primers, and 10 ng of template plasmid. After 20 cycles of site-directed mutagenesis PCR, the template plasmid was digested using DpnI restriction endonuclease (NEB, USA). Afterward, the PCR product was transformed to *E. coli* DH5a competent cells (Transgen, PR China). Single clones were selected and sequenced. All plasmids were extracted using QIAGEN Plasmid Maxi Kit (Qiagen, USA). To avoid the introduction of contamination in cell culture during the generation of pseudotyped viruses, extracted plasmids were inactivated in a 65 °C water bath for 30 min. The concentrations of plasmids were quantified using Nanodrop2000 (Thermo Scientific, USA).

### Pseudotyped virus package

First, we rescued the VSVΔG*-G virus based on methods developed in a previous publication [27]. In detail, HEK-293T cells with 80% confluence were co-transfected with pVSV-eGFP-dG, pCAG-VSVN, pCAG-VSVP, pCAG-VSVL, pCAG-VSVG, and pCAG-T7pol plasmids with a ratio of 10, 3, 5, 1, 3, and 5 μg, respectively, using Lipofectamine 3000 (Invitrogen, Lithuania), following the manufacturer’s guidance. The supernatant fluid was harvested 48 h after transfection, and one-half of the fluid was assigned to infect a second plate of HEK-293T cells transfected with pCAG-VSVG plasmid. The HEK-293T cells were examined by fluorescence microscopy for EGFP expression 24 h after infection. Titration of the VSVΔG*-G virus was quantified by the infection of HEK-293T cells, followed by flow cytometry analysis for an EGFP positive signal.

Second, we constructed pseudotyped viruses incorporated with S proteins from SARS-CoV, MERS-CoV, and SARS-CoV-2 using a protocol reported in previous research [28]. HEK-293T cells with 70–90% confluence were transfected with 15 μg of plasmids encoding SARS-CoV, MERS-CoV, and SARS-CoV-2 S proteins using Lipofectamine 3000. Simultaneously, plasmid encoding VSV-G proteins were transfected or mock-transfected into 293 T cells as a positive or negative control. The transfected cells were cultured at 37 °C with 5% CO_2_. 24 h later, the cells were infected with VSVΔG*-G viruses at MOI = 3–4 and incubated for 2 h. Afterward, the cell supernatant was discarded, and cells were gently rinsed with warm PBS twice. Then, 10 ml of fresh DMEM supplemented with 10% FBS, a 1% Penicillin-Streptomycin solution, and 20 mM HEPES was added to the dishes, and incubated at 37 °C with 5% CO_2_. 24 h later, these cells were checked for an EGFP positive signal, and the supernatant fluid was collected, centrifuged, filtered, and divided into aliquots. All of the pseudotyped viruses were stored at −80 °C. Repeated freezing-thawing cycles were avoided.

Huh-7 cells were used for the quantification of generated pseudotyped viruses. In detail, Huh-7 cells were cultured one day before quantification. Once 80% confluence was reached, the cells were digested, quantified, and seeded into a 48-well plate. After overnight incubation, the cells were infected by a series of 100, 50, 25, 12.5, 6.3, 3.2, and 1.6 μL of pseudotyped viruses with three replicates. The infected cells were placed at 37 °C with 5% CO_2_. 24 h post-infection, these cells were examined using a fluorescence microscope, followed by flow cytometry quantification for EGFP-positive cells. Pseudotyped viruses were titrated (transducing units, TU) by counting individual cells infected by the pseudotyped viruses. The final concentration of VSVΔG*-SARS2, VSVΔG*-SARS, and VSVΔG*-MERS was 4.85 × 10^6^ TU/mL, 5.54 × 10^6^ TU/mL, and 1.14 × 10^7^ TU/mL, respectively.

### Infection of cell cultures by VSV pseudotyped viruses

To enhance the reproducibility of this research, P3 and P4 passages of primary cell cultures were used for in vitro infection of pseudotyped viruses. Cell cultures were seeded into 48-well plates (10,000 cells per well), cultured in DMEM-F12 supplemented with 10% FBS, a 1% penicillin–streptomycin solution, and 20 mM HEPES. When the cells reached 70% confluence, culture supernatants were discarded. The cells were rinsed twice using a warm PBS buffer supplemented and a 1% penicillin–streptomycin solution. The cell cultures were infected by pseudotyped viruses, including pseudotyped viruses bearing wild-type and mutated S proteins, VSV-G glycoproteins, and negative control (culture supernatant harvested from mock-transfected cells). Each infection experiment had three independent replicates. The viruses were maintained in the culture medium supplemented with 1% FBS and a 1% penicillin–streptomycin solution for 24 h, followed by microscope observation and flow cytometry analysis for GFP positive cells. For VSVΔG*-SARS and VSVΔG*-SARS2, cell cultures were infected at MOI = 0.15 (referred to Huh-7 cells). For VSVΔG*-MERS, cell cultures were infected at MOI = 0.3 (referred to Huh-7 cells). No correlation was observed between the transduction rates of VSVΔG*-noG and VSVΔG*- SARS2 (cor = −0.0637, *p* > 0.05), between VSVΔG*-noG and VSVΔG*-SARS (cor = −0.0324, *p* > 0.05), and between VSVΔG*-noG and VSVΔG*-MERS (cor = −0.0723, *p* > 0.05).

Because the infection dosage that transduces the same percentage of cells doesn’t reflect the same number of viral particles, and the same amount of viral particles of pseudotyped viruses bearing different mutated S proteins does not infect cells with the same efficiencies, it is difficult to adjust pseudotyped viruses into the same infection dosage when cells are infected by pseudotyped viruses bearing mutated SARS-CoV-2 S proteins. And, although we attempted to quantify pseudotyped viruses by RT-qPCR, we found the results contained high systematic errors due to 1) the lack of reasonable reference genes; 2) the fact that the efficiencies of RNA extraction vary among different viruses; 3) the error induced by qPCR technologies; and 4) the yield concentration of pseudotyped viruses was not ideally stable. Therefore, we used a volume of 75 μL pseudotyped viruses to infect each cell culture in each cell infection test.

### Flow cytometry analysis

Cells infected or mock-infected with pseudotyped viruses were observed by a fluorescent microscope. After the culture medium was discarded, cells were rinsed twice in warm PBS, followed by 0.25% EDTA-trypsin digestion for 5 min. Then, the cells were collected into 1.5 mL tubes. The cells were washed twice with PBS to remove trypsin, and filtered using a 70 μm cell strainer (BD Falcon, USA) to remove clumps of cells. The final cells were placed on ice in a dark chamber. Flow cytometry analyses were performed (CytoFLEX, Beckman Coulter, USA). Cells infected with VSV△G*−G viruses or negative control were used to optimize the voltage for FSC, SSC, and FITC detectors, and to quantify the cell populations for positive or negative particles. For each infection assay, at least 5000 total cells were inputted. The percentage of transduction rates was displayed as mean ± SD based on three independent replicates.

### Molecular dynamic simulations and interaction prediction

The interface between the RBD of the S protein of SARS-CoV-2 and ACE2 orthologs from different species was examined by molecular dynamic (MD) simulations using Amber 20 [29**–**31]. We first downloaded the amino acid sequences from the NCBI (National Center for Biotechnology Information) database or our generated RNA-seq dataset. The RBD-ACE2 crystal structure (PDB ID: 6M0J) was downloaded from the Protein Data Bank [32], and served as template to prepare 3D models for SARS-CoV-2 S-RBD and ACE2 orthologs on SWISS-MODEL [33]. Next, each system was checked by leap and solvated in a cubic periodic box of TIP3P water extended by 10 Å from the solute. The system was neutralized using a rational number of counter ions of Na^+^ or Cl^−^, followed by parameterization using an Amber ff14SB force field [34]. Next, 10,000 steps of energy minimization including 5000 steps using the steepest descent method and 5000 steps of conjugate gradient minimization were performed. Each system was then heated to 300 K in the NVT ensemble by 0.2 ns. The minimization, heating, and equilibrium simulations were performed with strong constraints (500 kcal/mol/Å^2^) on heavy atoms with the sander program in Amber20. The 30 ns MD simulations were performed under constant temperature at 300 K with NPT ensemble and *pmemd.cuda*. The system temperature was maintained using Langevin dynamics. The cutoff distance between the Van der Waals energy and short-range electrostatic energy was 10 Å. The particle mesh Ewald method was used to calculate long-range electrostatic interactions. At least 3000 snapshots were extracted from the equilibrium trajectory for the final average structure of each RBD–ACE2 complex. The MM/GBSA method was used to calculate the binding free energy (Δ*G*), and the binding free energy was decomposed into the energy contribution of each residue [35].

We explored whether any spike variant interacted with a specific ACE2 residue based on the transduction rate across species. For each spike variant, the cell lines/species were classified into two groups (high and low infected groups, respectively) using a k-means clustering method. Then, at each position of 42 key residues of ACE2 estimated by MD simulation, the amino acid with the highest number was identified as the major residue for that position, and its frequency was calculated. Lastly, a Fisher’s exact test was employed to quantify whether the major residue was evenly distributed between the high and low-infected groups. A residue with a Fisher’s exact test *p* value less than 0.05 indicates that the frequency of the residue significantly differed between high and low infection groups. This implies that it may have interacted with a spike variant.

### RNA-seq sequencing, orthologous gene sets, and gene expression profiles

To further improve the reproducibility of this study, we performed RNA-seq analysis for cell cultures that were generated. Total RNAs of the cell cultures that were used for in vitro infection analysis were isolated using TRIzol regent according to the users’ guide (Invitrogen, USA). RNA quality and concentration were assessed using an Agilent 2100 Bioanalyzer (Lexington, USA) prior to library construction. For each RNA sample, a library size of 150 bp was constructed using the library preparation kit NEBNext Ultra RNA Library Prep Kit for Illumina® (NEB, USA). Paired-end sequencing was performed using Novogene Co. Ltd on the “Illumina” NovaSeq 6000 platform. This generated ~2.7 billion reads. We used IllQC_PRLL.pl in the package “NGSQCTools” to remove low-quality reads of raw data [36]. To generate the species-specific ortholog sets and calculate expression values, we downloaded the reference genomes of species (if available) from the public database. For those without a reference genome, Trinity was used to perform de novo assembly of transcripts [37] based on previously generated liver, kidney, and brain transcriptome to assemble transcripts [38]. The fixed parameter k-mer size was 25, the minimum contig length was 200, and the paired fragment length was 500. We then used “cd-hit-est” to process the assembled transcripts from different tissues of the same species, cluster the sequences with 90% similarity, and leave the longest transcript in each cluster [39, 40]. Finally, Augustus was employed to perform gene prediction on the de-redundant transcripts and to obtain GTF annotation files.

To construct the human reference sequence, we used “gffread” in the “cufflink” package to extract the human CDS sequence, filtered out incomplete ORF transcripts and pseudogene transcripts, and extracted the longest transcript for each gene [41]. BLAST was used to remove highly repetitive and highly similar genes with e-value <10^−6^ and Identity >90% as the filtering threshold [42]. The mapping rates across the dataset ranged from 9.63% to 66.58%. Finally, 18,552 unique protein-coding genes were obtained as reference sequences.

The longest transcript of each gene was extracted and reciprocal “BLAST” was performed with the protein sequences from the human samples [43]. The filtering threshold was set at 10^−6^ for e-values and 30% for identity. Two genes that were best aligned with each other were defined as orthologous genes. Because the complete genome and the de novo genome are quite different when compared, we used the CDS sequence of orthologous genes as the reference genome and generated annotation files in GTF format for RNA-seq data mapping. STAR was used to construct an index based on the sequence size of the orthologous gene set and the read length of different species. We used the default parameters to align the RNA-seq data with the reference genome [44]. We used “featureCounts” in the Subread software package to count reads, and eliminate multiple-matched reads [45].

Finally, we calculated the library size of each sample as a normalization factor. The R software package “edgeR” was used to normalize the library size and gene length (based on humans) by log_2_(RPKM-TMM + 1) [46, 47]. The sample information, mapping rates, list of orthologs, gene length, and expression levels are available in Tables S12–S17.

### Regression analysis and gene set enrichment

In order to identify genes whose expression is associated with the transduction of VSVΔG*-SARS2, VSVΔG*-SARS, and VSVΔG*-MERS, a generalized linear model was used to test the correlation between gene expression and transduction rates. If one orthologous gene was not identified in a particular species, that gene was assumed to not be expressed in that species (gene expression value = 0). We filtered out genes that were expressed in less than three samples. The ordinary least squares, Brownian model, and Ornstein-Uhlenbeck model were used to determine the best correlation in the transduction rate of each gene. Because a phylogenetic signal was not detected for the transduction rates of VSVΔG*-SARS2 and VSVΔG*-SARS, phylogenetic relationships were not considered in the regression analyses of transduction rates of the two pseudotyped viruses. However, a two-step method was employed in all regression analyses to correct the *p* value [47, 48]. In the first step, the species that had the greatest impact on the slope (i.e., potential outliers) was removed by the residuals, and then the regression was performed again. We defined the resulting *p* value as *p*.*robust* to remove the influence of the outliers on the regression. The second step was to repeat the regression process for the remaining species and remove one of remaining species each time until all remaining species were removed once. We then scored the largest (least significant) *p* value in the process as *p.max* to remove the impact of species on the regression. The cutoff of significant genes was set at *p*.*robust* < 0.01 and *p*.*max* < 0.05. Gene enrichment analyses were performed using DAVID 2021 updated (Database for Annotation, Visualization and Integrated Discovery) [49]. The parameter for EASE was 0.3 with Fisher’s exact test.

### Statistical analysis

R was used for plotting and statistical analysis; the values were expressed as mean ± SD. When cell cultures were infected by the VSVΔG*-SARS2 bearing the wildtype S protein, the relative transduction rates were classified into four categories (i.e., minimal-, slight-, moderate-, and efficient transduction) based on the following principles. First, we listed the quantile of all transduction rates and calculated the interquartile range (IQR). Then, we calculated the standard deviation (SD) of the transduction rates ranging from minimum to Q1. This was named SD_Q1._ A cell infection with a transduction rate of less than ten times SD_Q1_ was classified as a negligible infection; a cell infection with a transduction rate between 10* SD_Q1_ to Q3 was classified as a slight infection; a transduction rate between Q3 to Q3 + 1.5*IQR was classified as a moderate infection; and a transduction rate of more than Q3 + 1.5*IQR was classified as an efficient infection. This principle also was applied to evaluate the infection results of cell cultures by VSVΔG*-SARS and VSVΔG*-MERS. When cell cultures were infected by VSVΔG*-SARS2-Smut pseudotyped viruses bearing mutated S proteins from SARS-CoV-2, the infection results of each VSVΔG*-SARS2-S_mut_ were normalized to the relative transduction results of A549 cells. The outliers were postulated as mutations that significantly enhanced the tropism of SARS-CoV-2.

## Results

### In vitro infection assay and transduction rate of VSVΔG*-SARS2

**Fig. 1:**
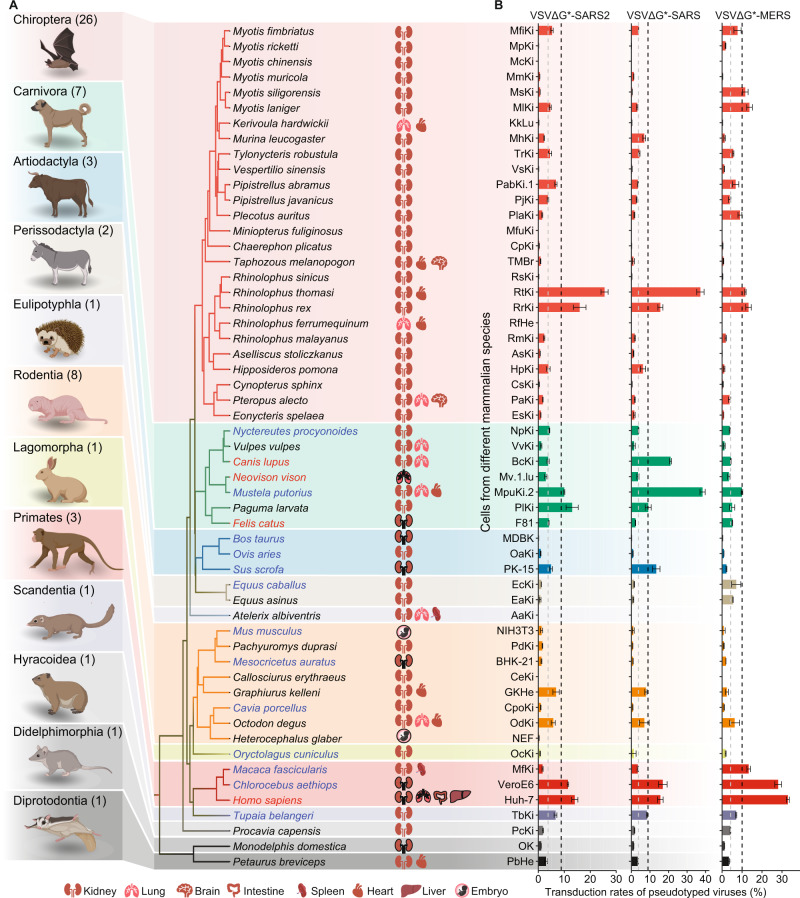
Transduction rates of SARS-CoV, MERS-CoV, and SARS-CoV-2 pseudotyped viruses to mammalian cell cultures. **A** Phylogenetic tree of 55 mammalian cell cultures was used in this study. A phylogeny was retrieved from TIMETREE (http://www.timetree.org/). Taxa indicated in red font are species that can be naturally infected by SARS-CoV-2 based on previous studies, while taxa indicated in blue font are species that were tested by in vivo experimental infection analyses. Moreover, 83 cell cultures were derived from the kidneys, heart, lungs, brain, and other tissues. The cartoon elements with black frames are cell lines derived from these tissues. The tiny cartoons were obtained from BioRender (https://biorender.com/). **B** Transduction rates (mean ± SD, *n* = 3) of 83 cell cultures infected by VSVΔG*-SARS2, VSVΔG*-SARS, and VSVΔG*-MERS viruses. Some cell cultures were derived from the same host. In that case, cell cultures with the highest transduction rates are displayed in this panel. The gray and black dashed lines represent the standard to define moderate and efficient transduction, respectively.

To assess susceptibility to SARS-CoV-2, SARS-CoV, and MERS-CoV, GFP-encoding pseudotyped viruses bearing relevant S proteins, named as VSVΔG*-SARS2, VSVΔG*-SARS, and VSVΔG*-MERS, respectively, were generated (Fig. S1). Then, we collected cell cultures derived from 55 mammalian species belonging to 12 orders, including Diprotodontia (one species), Didelphimorphia (one species), Hyracoidea (one species), Scandentia (one species), Primates (three species), Lagomorpha (one species), Rodentia (eight species), Eulipotyphla (one species), Perissodactyla (two species), Artiodactyla (three species), Carnivora (seven species), and Chiroptera (26 species) (Fig. 1, Fig. S1). A total of 83 cell cultures, including 64 primary cell cultures, three immortalized cell cultures, and 16 cell lines were obtained (Table S1). Of these cell cultures, 56 were derived from the kidneys in which ACE2s were abundantly expressed; 10 were derived from the lungs; seven were derived from the heart; and the 10 remaining cell cultures were derived from other tissues, such as the embryo, colon, and spleen (Fig. 1, Table S1). These cell cultures were infected by pseudotyped viruses and subjected to flow cytometry analysis of GFP-positive signals to quantify their transduction rates (Fig. S1). Overall, the cell cultures derived from the kidneys showed relatively higher transduction rates than those from other tissues (Fig. S2A). No significant differences in transduction rates to pseudotyped viruses were observed between primary cell cultures and permanent cell lines (Fig. S2B).

Upon infection with VSVΔG*-SARS2, the 83 cell cultures displayed different sensitivities, with an overall transduction rate of 0–26.7% (Fig. 1, Fig. S3, Table S1). Forty-four cell cultures were minimally transduced by VSVΔG*-SARS2 (transduction rate of <1.2%). A total of 32 cell cultures were slightly (transduction rate of 1.2–3.8%) or moderately (transduction rate of 3.8–8.8%) transduced by VSVΔG*-SARS2, including cell cultures derived from dogs (BcKi, BcLu, and MDCK), cats (F81), ferrets (MpuHe), raccoon dogs (NpKi), minks (Mv.1.Lu), tree shrews (TbKi), and pigs (PK-15) (Fig. 1, Fig. S3). This is consistent with the fact that raccoon dogs, dogs, cats, minks, ferrets, tree shrews, and hamsters are known to be susceptible to SARS-CoV-2 with asymptomatic to moderate clinical signs (Table S2) [22, 50]. We found that VSVΔG*-SARS2 can infect PK-15 cells (4.9%) (Fig. 1), but inconsistent conclusions were drawn by previous experimental infections. Several studies showed that pigs were resistant to SARS-CoV-2 [23, 51, 52], while other studies showed that SARS-CoV-2 could infect pigs and be transmitted to co-housed naive sentinel pigs [53, 54]. Cell cultures from several pets, such as the heart (OdHe), lung (OdLu), and kidney (OdKi) cells of the common degu (*Octodon degus, a hystricomorph rodent*) and heart (GkHe) cell cultures of Kellen’s dormouse (*Graphiurus kelleni, a glirid rodent*) were moderately transduced by VSVΔG*-SARS2 (Fig. 1). Mice were found to be resistant to the SARS-CoV-2 infection; however, cell cultures from mice (NIH3T3 and SP2/0) were slightly transduced by VSVΔG*-SARS2 (Fig. S3, Table S1). Given that SARS-CoV-2 can adapt by serial passaging in the respiratory tract of aged BALB/c mice [55, 56], we surmised that animals who were not susceptible to SARS-CoV-2 could be potential hosts following viral evolution during repeated exposures.

Lastly, seven cell cultures derived from Thomas’s horseshoe bats (RtKi), king horseshoe bats, humans, palm civets, Africa green monkeys, and ferrets were found to be highly susceptible to VSVΔG*-SARS2 (transduction rate of >8.8%). Among them, humans, palm civets, and ferrets were identified as susceptible hosts for SARS-CoV and SARS-CoV-2 based on in vivo analyses (Table S2) [57]. In particular, 14.0% of Huh-7 (human cell line), 11.4% of Vero-E6 (green monkey cell line), 11.9% of Marc-145 (green monkey cell line), and 10.0% of MpuKi.2 (ferret kidney cell line) were transduced, which confirmed that these cell lines are highly susceptible to SARS-CoV-2, as found in previous studies [28, 58]. We noted that palm civet kidney cells (PlKi) were highly susceptible to VSVΔG*-SARS2, as palm civets have been recognized as one of the replication hosts of SARS-CoV [8, 59] (Fig. 1, Fig. S2). Importantly, ferret (*Mustela putorius furo*) kidney cells (MpuKi.2) also were highly susceptible to SARS-CoV-2. Experimental studies have shown that ferrets are susceptible to SARS-CoV-2 [23, 51, 60–62]. Primary cells cultures derived from Thomas’s horseshoe bat and the king horseshoe bat were found to be more sensitive to VSVΔG*-SARS2 than the human Huh-7 cell line (Fig. 1, Table S1), suggesting they may serve as likely hosts for SARS-CoV-2.

### Host tropism of VSVΔG*-SARS and VSVΔG*-MERS

The maximum likelihood estimates of Pagel’s *λ* for the transduction rates of VSVΔG*-SARS2, VSVΔG*-SARS, and VSVΔG*-MERS were 6.74 × 10^−5^ (phylogenetic signal test: log_λ_ = −163.95, *p* > 0.05), 6.33 × 10^-5^ (log_λ_ = −192.22, *p* > 0.05), and 0.74 (log_λ_ = −174.13, log_0_ = 1475.26, *p* = 0.09 × 10^−2^), respectively, indicating that closely-related taxa generally have similar susceptibility to VSVΔG*-MERS but not to VSVΔG*-SARS and VSVΔG*-SARS2. Consistently, VSVΔG*-SARS showed a more similar tropism profile with VSVΔG*-SARS2 (*R*^2^ = 0.61, *p* < 0.001, phylogenetic generalized least squares test) than with VSVΔG*-MERS (*R*^2^ = 0.29, *p* < 0.001, phylogenetic generalized least squares test). In particular, the 83 cell cultures infected by VSVΔG*-SARS displayed a transduction rate of 0–38.4% (Fig. 1). Overall, 38 cell cultures were minimally (transduction rate of 0–1.1%), 23 slightly (1.1–3.8%), 10 moderately (3.8–8.9%), and 11 highly (8.9–38.4%) transduced by VSVΔG*-SARS. Cell cultures that showed the highest susceptibility to VSVΔG*-SARS were derived from domesticated ferrets, with 38.4% of ferret kidney MpuKi.2 cells and 31.1% of ferret MpuKi.1 cells transduced. This represents a 2.6-fold and 2.0-fold increase compared to that of human cells (Huh-7) (Fig. 1, Fig. S4, Table S1). This is noteworthy as ferrets have been used as an animal model for SARS-CoV and experience clinical signs, including sneezing, fever, and diarrhea [63]. The cell culture that ranked highest after ferret kidney cells was derived from Thomas’s horseshoe bat, which had a transduction rate of 37.2%, indicating that this species might also serve as an important host for SARS-CoV.

Palm civets have been recognized as a replication host for SARS-CoV. Our results indicate a consistent and high transduction rate (9.1%) for palm civet PlKi cells. In addition, VSVΔG*-SARS displayed a higher capacity than VSVΔG*-SARS2 in transducing cell cultures from highly susceptible species, such as RtKi (37.2% vs 25.5%), PK-15 (13.4% vs 4.9%), MpuKi.2 (38.4% vs 10.0%), MpuKi.1 (33.3% vs 1.9%), and BcKi (21.1% vs 3.8%) cells (Fig. 1, Fig. S4). This implies that SARS-CoV might be easier to transmit from humans to natural hosts than SARS-CoV-2.

We found that 41 cell cultures were minimally (transduction rate ranging from 0 to 1.3%), 21 slightly (1.3–4.3%), 13 moderately (4.3–10.0%), and 8 efficiently (10.0–32.9%) transduced by VSVΔG*-MERS (Fig. 1, Fig. S3). Strikingly, human cell lines showed the highest transduction rate upon VSVΔG*-MERS infection, which was different than from VSVΔG*-SARS and VSVΔG*-SARS2. Moreover, among the top 10 cell cultures with the highest transduction rates, six (MlKi, RrKi, RtKi, MsKi, PlaKi, and MfiKi) were derived from bats (Fig. 1, Fig. S3), suggesting the several bat species serve as potential reservoir hosts for MERS-CoV. In particular, the Japanese pipistrelle bat (*Pipistrellus abramus*) is a species of concern. They live in close proximity to human communities and have the potential to transmit the virus to humans [64]. And, although previous studies have suggested that ferrets are resistant to MERS-CoV infection [65], we found that 10.0% of ferret kidney MpuKi.2 cell cultures were transduced by the MERS-CoV pseudotyped virus (Fig. 1). Moreover, the MERS-CoV receptor *DPP4* [66] is slightly expressed in ferret kidney cells. These results imply that there are factors other than DPP4 that can aid in MERS-CoV entry and that ferrets are susceptible to SARS-CoV, SARS-CoV-2, and MERS-CoV.

### SARS-CoV-2 S protein variants alter the susceptibility of animals

**Fig. 2:**
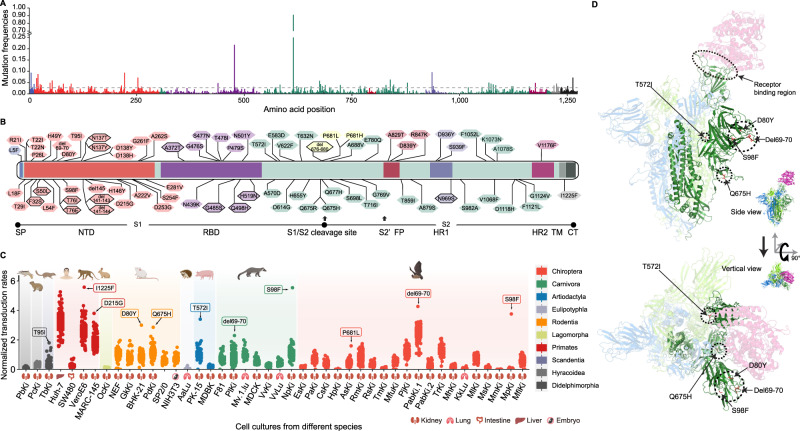
Characterization of spike variants that impact the host tropism of VSVΔG*-SARS2 across mammals. **A** The substitution frequency for each site was analyzed based on S sequences retrieved from the GISAID database. **B** Distribution of 79 site mutations from the S protein. The site with a black frame means this mutation was selected based on comparative analysis of the viral genome derived from animal hosts. **C** The overall normalized transduction rates of all mutations across different cell cultures. The tiny cartoons shown in the figure represent species from different mammalian orders. The tissue origin of these cell cultures is shown on the *x*-axis. **D** A 3D structure of the S-ACE2 complex showing that del69-70, D80Y, S98F, T572I, and Q675H are located outside the RBD of the S proteins.

To explore whether SARS-CoV-2 S protein variants alter the susceptibility to SARS-CoV-2, 77,125 spike genes of SARS-CoV-2 were retrieved from GISAID (retrieved on 2020/10/26), and 65 variants with residue frequencies higher than 0.075% were selected (Fig. 2A). In addition, fourteen residue mutations in S proteins from SARS-CoV-2 variants isolated from mouse, cat, dog, mink, and tiger samples were selected, as well as S proteins mutations from bat-CoV RaTG13 (GISAID: EPI_ISL_402131), bat-CoV RmYN02 (GISAID: EPI_ISL_412977), and pangolin-CoV MP789 (GISAID: EPI_ISL_412860). In total, 79 pseudotyped viruses with mutated SARS-CoV-2 S proteins were constructed, followed by the in vitro infection of 44 cell cultures (Fig. 2B).

We found that most of these mutations had little impact on the transduction efficiencies of VSVΔG*-SARS2-S_mut_ pseudotyped viruses to cell cultures (Fig. 2C, Fig. S5). As expected, cell cultures from humans, monkeys, and bats (Huh-7, Vero-E6, Marc-145, and PabKi.1) were more sensitive to pseudotyped viruses compared to other cell cultures (Fig. 2C). Previous studies have shown that substitutions N439K, N501Y, D614G, and H655Y in S proteins enhance the pathogenicity and transmission of SARS-CoV-2 in humans [67**–**69]. In the present study, we found that these substitutions do not always promote the transduction rate. For example, H655Y increased the transduction of VSVΔG*-SARS2 to tree shrew cells (TbKi) (Fig. S5), while the D614G substitution significantly reduced the capacity of VSVΔG*-SARS2 to transduce in naked mole-rat fibroblast cells and in Japanese pipistrelle bats cells (PabKi.1) (Fig. S6). In addition, the T22I substitution significantly reduced the tropism of SARS-CoV-2 to F81 (cat kidney cell line) and BHK-21 (golden hamster kidney cell line). Moreover, the H519N and T716I substitutions showed a decreased capacity to transduce PK-15 (pig kidney cell line) and PaKi (black flying fox kidney cell line). Finally, substitutions T29I, E281V, and S939F significantly reduced the tropism of SARS-CoV-2 to MfuKi (eastern bent-wing bat kidney cell) cell cultures (Fig. S5).

Several mutations were found to significantly increase the transduction efficiencies of VSVΔG*-SARS2-S_mut_ to certain cell cultures (Fig. 2C, Fig. S5). In particular, del69-70 in the S protein promoted the capacity of VSVΔG*-SARS2-S_del69-70_ to transduce bat PabKi.1 cells (17.8%) to a level twice that of Huh-7 cells (9.8%) (Fig. S5B). Similarly, VSVΔG*-SARS2-S_D80Y_ caused a 7.2% transduction rate in golden hamster cells, which was higher than that of Huh-7 cells (6.6%) (Fig. S5C). We found that substitution S98F significantly enhanced the transduction rate in raccoon dog kidney cells (62.4%) and Rickett’s big-footed bat kidney cells (45.0%) compared to Huh-7 cells (38.9%) (Fig. S5D). Similarly, substitution T572I significantly increased the transduction efficiency of VSVΔG*-SARS2 to pig PK15 cells (11.3%) compared to Huh-7 cells (12.2%) (Fig. S5E). The Q675H mutation also significantly enhanced the ability of the SARS-CoV-2 pseudotyped virus to infect fat-tailed gerbil kidney cells, with a transduction rate of 13.4% compared to 15.7% in Huh-7 cells (Fig. S5F). Notably, these five mutations were located outside the receptor-binding domain (RBD) of the S protein (Fig. 2D). Thus, these results support the idea that different mutations have a unique impact on viral susceptibility in particular mammalian species, and that variants within and outside the RBD region are of equal importance for viral entry and binding.

Molecular dynamic simulations using Amber 20, as well as the crystal complex of RBD-hACE2 (PDB ID: 6M0J), indicate that the binding free energy (Δ*G*) between RBD and different ACE2 orthologs ranged from −64.7 to −24.8 kcal/mol. ACE2s from green monkeys (*Chlorocebus sabaeus*) and greater horseshoe bats (*Rhinolophus ferrumequinum*) showed the highest and lowest binding free energy with SARS-CoV-2 S-RBD, respectively (Fig. S6A). We further analyzed the free energy contributions based on a per-residue basis (Fig. S6B–D). The results showed that there were 42 amino acid residues that were important to the interactions between RBD and ACE2s (Fig. S6B–E).

In this regard, the cross-species infection spectrum of different spike variants allowed us to explore the possible site-wise “interaction” between ACE2 amino acid residues and spike variants that can affect the transduction rate across species. By testing whether the frequency of the major residues was evenly distributed between high and low infection groups suggested by each spike variant, we identified 98 combinations of spike variants and ACE2 residues that were candidates for such potential “interactions” (“material and methods”, Fig. S7, Table S3). The top-ranked ACE2 positions involved in these significant interactions were 49, 31, 354, 82, 75, 35, 353, whereas the top-ranked spike variants included P26L, S254F, H519N, H146Y, A262S, and T478I. The majority of these interactions (86.7%) involved the spike variants of the RBD region (Table S3). These results support the possibility that different spike variants have a unique impact on viral susceptibility by interacting with distinct ACE2 residues and/or other intrinsic factors in particular mammal species.

### Genes with expression levels associated with transduction rates across species

**Fig. 3:**
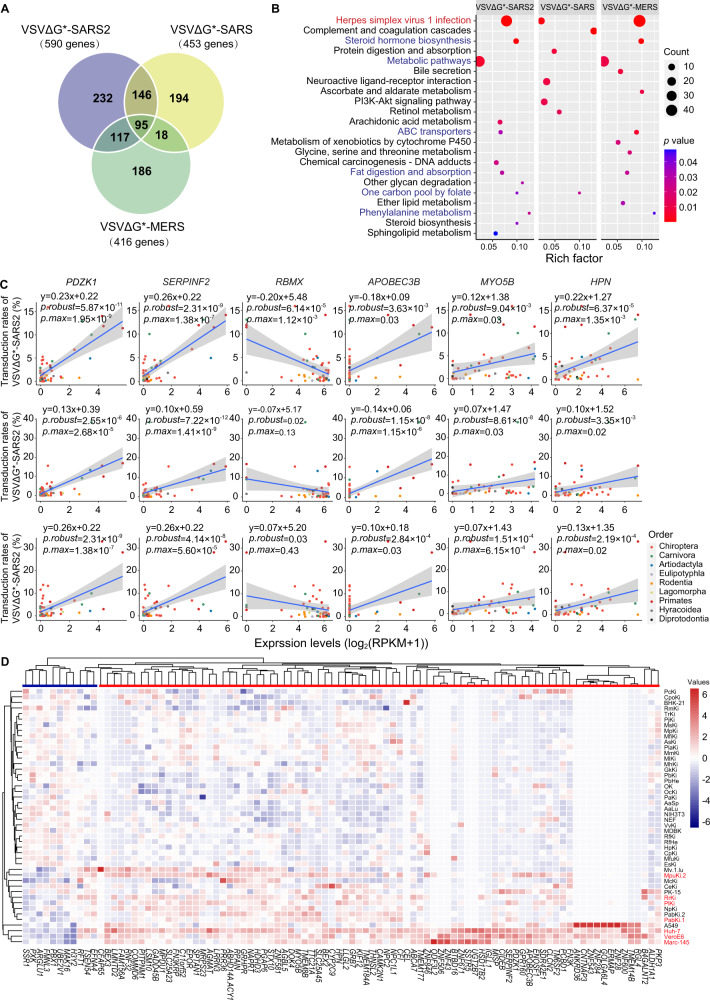
Comparative transcriptomics analyses identifying genes whose expression levels were correlated with the transduction rates of SARS-CoV, MERS-CoV, and SARS-CoV-2 pseudotyped viruses. **A** A Venn graph showing 590 genes, 453 genes, and 416 genes that were significantly associated with the transduction rates of VSVΔG*-SARS2, VSVΔG*-SARS, and VSVΔG*-MERS pseudotyped viruses, respectively. A total of 95 genes were commonly associated with the three pseudotyped viruses. **B** The KEGG pathways that were enriched by these genes and had expressions associated with the transduction rates of VSVΔG*-SARS2, VSVΔG*-SARS, and VSVΔG*-MERS. **C** Plots of several genes whose expressions were associated with the transduction rates of VSVΔG*-SARS2, VSVΔG*-SARS, and VSVΔG*-MERS. **D** Heatmap of 95 genes that were commonly associated with the three pseudotyped viruses. The expression level was represented by log_2_(RPKM + 1). The cell cultures marked by red fonts were efficiently transduced by VSVΔG*-SARS2. Blue bars under the tree indicate genes that were negatively associated with VSVΔG*-SARS2 transduction, while the red bars indicate genes that were positively associated with VSVΔG*-SARS2 transduction.

Transcriptomic profiles of cell cultures from 42 mammal species were generated, and regression analyses were performed to identify genes whose expressions were significantly correlated with the transduction rates of VSVΔG*-SARS2, VSVΔG*-SARS, and VSVΔG*-MERS. In total, there were 590 genes whose expression levels were significantly associated with the transduction rates of VSVΔG*-SARS2 (Fig. 3A, Table S4). The most significantly enriched pathway was the Herpes simplex virus 1 infection (*p* = 4.30 × 10^−23^, Fisher exact test), in which 93.6% (59 of 63 genes) of associated genes are zinc finger proteins (Fig. 3B, Table S5). The corresponding enriched biological processes that contain zinc finger proteins are DNA-templated transcription regulation (*p* = 2.40 × 10^−12^, Fisher exact test) and RNA polymerase II promoter transcription regulation (*p* = 5.10 × 10^−10^, Fisher exact test) (Table S6). Zinc finger proteins appear to function in host-virus interactions and play multiple roles in viral replication by regulating host cell transcription profiles [70]. Many zinc finger proteins are positively correlated with VSVΔG*-SARS2 infection rates. This indicates that the transcription levels of host cells are important for SARS-CoV-2 entry, which usually depends on a mechanism called “cap snatching,” as observed in the influenza virus [71]. The top five genes that were associated with the transduction rates of VSVΔG*-SARS2 in cell cultures were *PDZK1* (*p.robust* = 5.87 × 10^−11^), *SERPINF2* (*p.robust* = 2.31 × 10^−9^), *SCG5* (*p*.*robust* = 3.51 × 10^−9^), *DEPP1* (*p*.*robust* = 4.57 × 10^−9^), and *ABCC6* (*p*.*robust* = 2.71 × 10^-8^) (Fig. 3C, Fig. S8, Table S4). These genes are worthy of further exploration. For example, *PDZK1* encodes a PDZ (PSD95/DLG/ZO-1) domain-containing scaffolding protein (named NHERF3), which belongs to a group of proteins that mediate cell-cell junctions and is involved in the coordination of a diverse range of regulatory processes. A previous study showed that SARS-CoV and the neurotropic rabies virus are linked to the PDZ-binding functions of their envelope proteins [72]. PDZK1 can interact with SR-B1 and facilitate hepatitis C virus entry [73]. Moreover, the ACE2 C-terminal PDZ-recognition motif ^802^QTSF^805^ binds to *NHERF1* and/or *NHERF3* and promotes ACE2-mediated SARS-CoV-2 cell entry [74, 75]. *SERPINF2* encodes one of the serine protease inhibitors. A recent study revealed that *SERPINF2* expression increases in serum levels of patients with COVID-19, along with several other SERPINs (*SERPINA1* and *SERPINA3*) [76]. In addition, several genes, such as *MAK16* (*p*.*robust* = 1.92 × 10^−5^), *H3Y2* (*p*.*robust* = 4.08 × 10^−5^), and *RBMX* (*p*.*robust* = 6.14 × 10^−5^), showed a significant negative correlation with transduction rates (Fig. S8, Table S4). The role of *MAK16* and *H3Y2* in viral infection is unclear, while *RBMX*, which encodes X-ed RNA binding motif proteins, responds to SARS-CoV-2 entry via viral RNA-host protein interactions [77, 78].

We identified a significant association between the expression levels of 453 genes associated with the transduction rate of VSVΔG*-SARS and 416 genes associated with the transduction rate of VSVΔG*-MERS. These genes are primarily enriched in metabolic pathways and Herpes simplex virus 1 infection, consistent with the fact that more than half of all gene expressions were correlated with VSVΔG*-SARS2 transduction (Fig. 3B, Tables S6–S11). Genes whose expressions were associated with VSVΔG*-MERS exhibited a similar profile to VSVΔG*-SARS2 in biological process enrichment, particularly in DNA-templated transcription regulation and RNA polymerase II promoter transcription regulation. However, the top biological process that was enriched by genes whose expressions were associated with VSVΔG*-SARS transduction was proteolysis (*p* = 7.7 × 10^−4^, Fisher exact test) (Table S8), highlighting that the proteases of host cells are essential for SARS-CoV pseudotyped virus infectivity [79]. Moreover, a recent study suggests that proteases are central to the infection process of SARS-CoV-2, and drugs targeting proteases can cause a dose-dependent reduction in SARS-CoV-2 titers [80]. This suggests that proteases could be a common target for treating SARS-like diseases. However, the expressions of recognized receptors (*ACE2* and *DPP4*) of SARS-CoV and MERS-CoV did not significantly correlate with transduction rates. Given this result, we examined the potential roles of those related genes. Several gene responses were found (e.g., *HSD17B2*, *IGLL1*) in SARS-CoV-2 infection [81, 82], whereas the role of top-ranked genes associated with VSVΔG*-SARS transduction was more limited.

We compiled a list of 95 genes that were commonly associated with the transduction rates of SARS-CoV, MERS-CoV, and SARS-CoV-2 pseudotyped viruses (Fig. 3D, Table S12). This list not only included genes mentioned above, but also included genes such as *APOBEC3B*, *MYO5B*, and *HPN* (hepsin), which may be involved in SARS-CoV-2 virus–host interactions. For example, *APOBEC3B*, which belongs to a family of proteins in mammals, consists of cellular cytosine deaminases and serves as a barrier that potentially prevents the cross-species transmission of lentiviruses [83]. In addition, *APOBEC3* can constantly shape the SARS-CoV-2 genome by editing cytidine to uridine [84]. In contrast, *MYO5B* is downregulated in a Spike-RBD-induced mast cell degranulation model and plays a role in endosomal transport [85]. *MYO5B* also interacts with viral proteins. A previous study suggests that inhibiting MYO5 proteins could be an effective target for COVID-19 treatment [85]. These results broadly implicate these common genes in viral entry and replication, and therefore they remain suitable targets for further evaluation of cross-species transmission risks.

## Discussion

Herein, we have demonstrated that SARS-CoV-2, SARS-CoV, and MERS-CoV can infect the cells of dozens of mammal species, indicating they are generalist viruses and not specifically adapted to humans. In addition, cell cultures from different mammals show variable susceptibilities to SARS-CoV-2, SARS-CoV, and MERS-CoV pseudotyped viruses. This implies that SARS-CoV-2 has the capacity to spillover to multiple species and establish natural reservoirs after minor or major adaptive evolutionary changes. Our results highlighted the potential for Thomas’s horseshoe bats, king horseshoe bats, green monkeys, and ferrets to serve as reservoir hosts for these coronaviruses. Specially, primary cells cultures derived from Thomas’s horseshoe bat and the king horseshoe bat were found to be more sensitive to VSVΔG*-SARS2 than human cell lines. This is important, as horseshoe bats (Rhinolophidae) are considered as a reservoir for many zoonotic viruses and they are assumed to be generally tolerant to infection. However, very rare sarbecoviruses have been reported in both Thomas’s horseshoe bats and the king horseshoe bats, but not in other horseshoe bats (e.g., Chinese horseshoe bats) [86]. This not only raises the concern that SARS-CoV-2 might spillover from humans to these two bat species, but also prioritizes the surveillance of virus intolerance in horseshoe bats.

Since ACE2 was found to be the functional receptor for the spike protein of SARS-CoV and SARS-CoV-2, pioneer studies have predicted the susceptibility of various animal species to SARS-CoV-2 using ACE2 sequences and/or their binding affinity to spike proteins [15, 87, 88]. While our experimental assay validated the susceptibility of several species (e.g., rhesus macaque and golden hamster) to SARS-CoV-2, there is a considerable inconsistency between those in silico predictions and our cellular assays. For example, the Chinese tree shrew and ferret were predicted to exhibit low or no risk for SARS-CoV-2 infection [15], possibly due to the fact that their ACE2 sequences diverged from human ACE2. However, our experiments indicated both species are of medium susceptibility. Similarly, bats are generally predicted to be of low infection risk, yet our results suggest that several bat species are likely to be highly infected. Thus, studies that experimentally examine infection susceptibility are extremely valuable for comparison with future in silico predictions. In addition, as indicated in Table S2, 75% of the in vitro susceptibilities presented in our study were supported by in vivo inoculation assays. Differences between in vitro and in vivo assays were observed in the mouse, red fox, and cow. For example, inoculation assay suggested that mice are resistant to SARS-CoV-2 infection, but our study found their cell cultures show moderate transduction rates. This may be due to the fact that cell models may fail to resemble the complexity of a whole organism, or to the particular challenges of translatability of in vitro-generated data into in vivo models [57]. Nonetheless, in vivo testing can benefit from in vitro testing in determining the susceptibility risk and natural hosts of SARS-CoV-2 and other sarbecoviruses.

We also examined the susceptibilities of several mammalian species to different spike variants and found that each spike variant showed a distinct spectrum of infection. Specially, several spike variants (Del69-70, D80Y, S98F, T572I, and Q675H) that are not in the RBD region were shown to increase the infectivity of SARS-CoV-2. The variant del69-70 was initially identified at the beginning of 2020, and over the past several years it has mutated into three different variants (i.e., Y453F, S493K, and N501Y) [89]. Previous reports have shown that del69-70 increases cleavage in the S protein and infectivity in the SARS-CoV-2 B.1.1.7 variant [90, 91]. This mutation may enhance SARS-CoV-2 spilling over to other animals, such as the Japanese pipistrelle bat, which inhabits urban areas of high human population density. In addition, an interaction between ACE2 residues and spike variants, which potentially affect infection rates, was found in our study. Some of these ACE2 residues are import for the interface between the SARS-CoV RBD and ACE2. For example, Lys31 and Lys353 are virus-binding hotspots, which consist of salt bridges between Lys31 and Glu35 and between Lys353 and Asp38 [92, 93]. However, most of the spike variants in these predicted interactions were located in the N-terminal domain (NTD). Thus, regions inside and outside of RBD may be of equal importance for viral entry and infection. Greater attention should be paid to these spike variants, as they (e.g., P26L, S254F, H146Y, and A262S) constitute the ‘NTD supersite’, which is recognized by all known NTD-specific neutralizing antibodies [94], and thus could mediate immune escape.

An examination of phylogenetic signals indicates that SARS-CoV exhibits a more similar tropism profile with SARS-CoV-2 than with MERS. This may be due to the fact that SARS-CoV and SARS-CoV-2 use the same receptor. However, the SARS-CoV pseudotyped virus had higher efficiency in transducing several cell cultures from dogs, pigs, and ferrets, suggesting that further investigation may uncover spike variants and other factors between SARS-CoV and SARS-CoV-2 that contribute to this difference (Fig. S4). Regression analyses indicated that the expression of commonly known receptors and factors known to facilitate the entry of SARS-CoV-2, SARS-CoV, and MERS-CoV, did not correlate with cross-species infection (Fig. S8). In particular, the expression levels of *ACE2* (*p*.*robust* = 0.14)*, FURIN* (*p*.*robust* = 0.89) and *TMPRSS2* (*p*.*robust* = 0.11), showed an insignificant correlation with VSVΔG*-SARS2 transduction rates (Figs. S9A–C; S9D, and Table S4, Table S6). In contrast, *CTSL* (*p*.*robust* = 7.4 × 10^−3^) was significantly correlated with VSVΔG*-SARS2 transduction rates.

We found that certain cell lines expressed high levels of *ACE2* (e.g., cells derived from the sugar glider, Nepalese whiskered bat, and greater horseshoe bat), but these were minimally or only slightly transduced by VSVΔG*-SARS2 (Fig. S9A-C and Table S1). However, this may not negate the role of ACE2 in determining SARS-CoV-2 susceptibility in other species because RNA expression cannot fully represent the ACE2 protein on the cell surface. Moreover, subsequent penalized regression model analyses revealed that sequence changes in ACE2s are significantly correlated with transduction rates across phylogenetically diverse taxa (*F* = 15.78, *p* = 8.9 × 10^−13^) (Figs. S10, S11). This suggests that changes in ACE2 sequences rather than expression contribute to differences in susceptibility. However, the total variance of transduction rates explained by ACE2 sequence changes ~28.9%. Therefore, future studies should explore other host intrinsic factors associated with susceptibility, such as genes identified in this study whose expression was significantly correlated with transduction rates.

Finally, our results highlight the fact that cell culture models represent an important method of understanding cross-species transmission of sarbecoviruses by identifying the spectrum of mammalian hosts that are susceptible to SARS-CoV-2, SARS-CoV, and MERS-CoV and their variants.

## Supplementary information

Supplemental figures

Table S1

Table S2

Table S3

Table S4

Table S5

Table S6

Table S7

Table S8

Table S9

Table S10

Table S11

Table S12

Table S13

Table S14

Table S15

Table S16

Table S17

## Data Availability

RNA sequencing data from this study have been deposited in ScienceDB (10.57760/sciencedb.j00001.00445) and in the Genome Sequence Archive in National Genomics Data Center, China National Center for Bioinformation/Beijing Institute of Genomics, Chinese Academy of Sciences (GSA: CRA009056) and NCBI (PRJNA906190).
