# Supplementary material for: Comparative susceptibility of SARS-CoV-2, SARS-CoV, and MERS-CoV across mammals
Source: ISME J. 2023 Jan 23;17(4):549–60. doi: 10.1038/s41396-023-01368-2 (PMC9869846; doi:10.1038/s41396-023-01368-2)
Supplement: Supplementary file 1 — Supplemental figures [file 41396_2023_1368_MOESM1_ESM.pdf]

Supplementary Information for

Comparative susceptibility of SARS-CoV-2, SARS-CoV, and MERS-CoV across mammals

Meng Li, Juan Du, Weiqiang Liu, Zihao Li, Fei Lv, Chunyan Hu, Yichen Dai, Xiaoxiao Zhang, Zhan Zhang, Gaoming Liu, Qi Pan, Yang Yu, Xiao Wang, Pingfen Zhu, Xu Tan, Paul A. Garber, Xuming Zhou

Correspondence to: zhouxuming@ioz.ac.cn

This PDF file includes:

Figs. S1 to S11

Other Supplementary Materials for this manuscript include the following (separate files):

Tables S1 to S17

Supplementary Figures

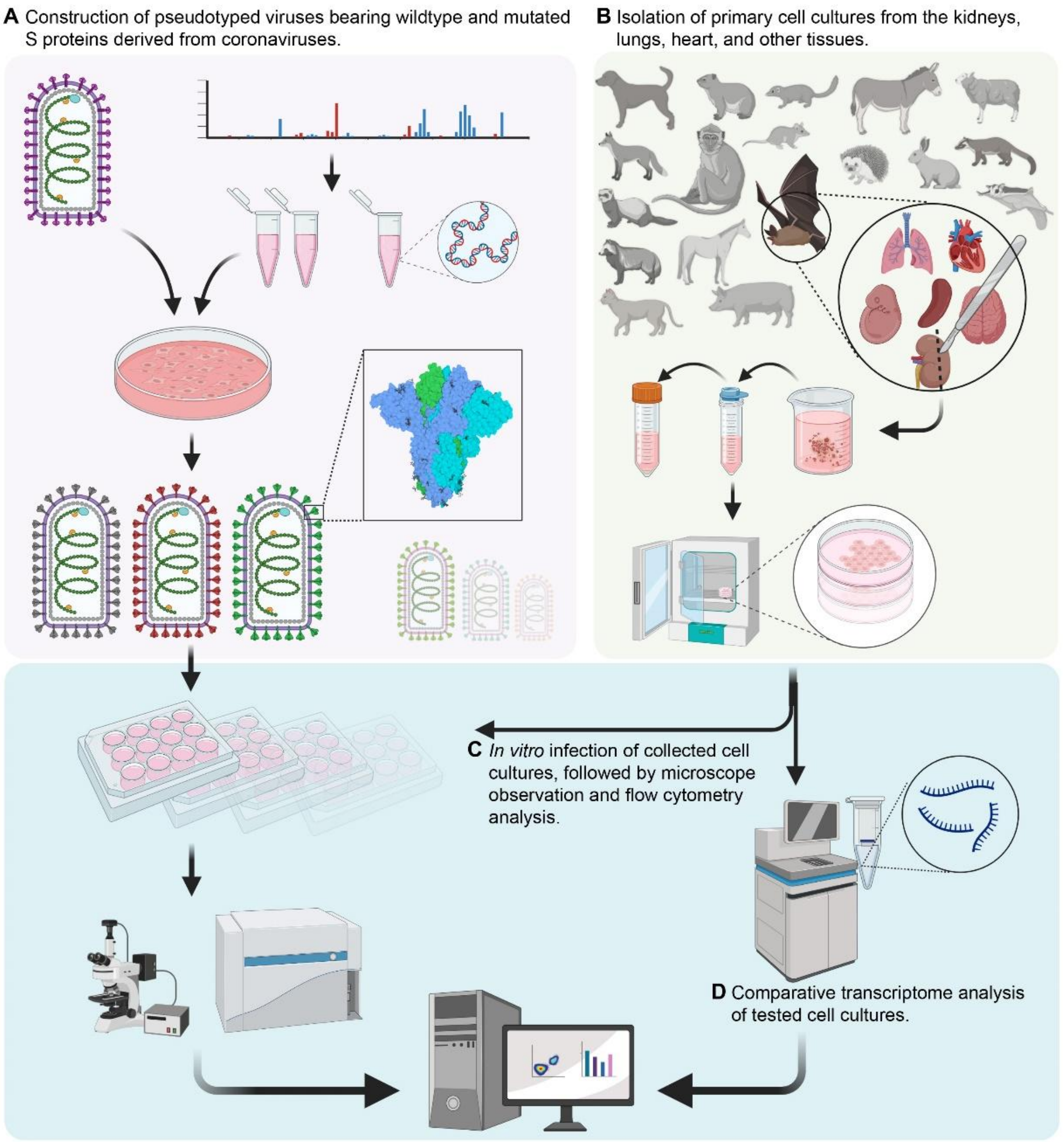

Fig. S1: Schematic description of this research. (A) VSV-ΔG*-G pseudotyped virus packaging system was adopted in this research. We constructed three pseudotyped viruses bearing SARS-CoV, MERS-CoV, and SARS-CoV-2 S proteins. We also constructed seventy-nine pseudotyped viruses bearing mutated SARS-CoV-2 S proteins. **(B and C)** These viruses were subjected to infect 83 cell lines or primary cell cultures derived from 55 mammals. **(D)** The transduction rates were quantified by flow cytometer, and analyzed together with transcriptome data.

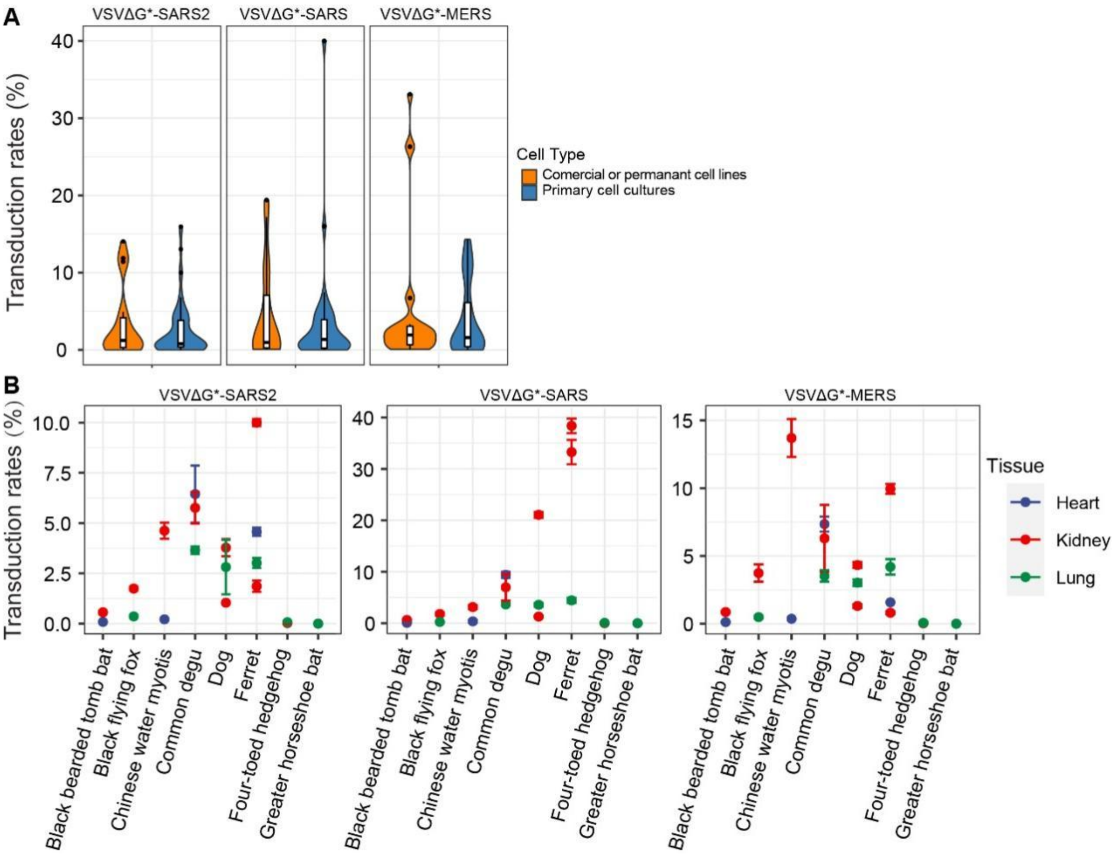

Fig. S2: Overview of transduction rates based on derived tissues and cell types. (A) Permanent cell lines or primary cell cultures were infected by SARS-CoV, MERS-CoV, and SARS-CoV-2 pseudotyped viruses without significant difference. **(B)** Cells cultures derived from kidneys are more susceptible to pseudotyped viruses than cells cultures derived from lung or heart tissues. From 8 species, we collected cell cultures from multiple tissues (i.e., kidneys, lungs, or hearts, brains, etc.). We found cell cultures from the kidneys are more susceptible to pseudotyped viruses, especially in black flying fox, Chinese water myotis, dog, and ferret kidney cells. Three independent replicates were set for each infection assay. The bar means Mean±SD.

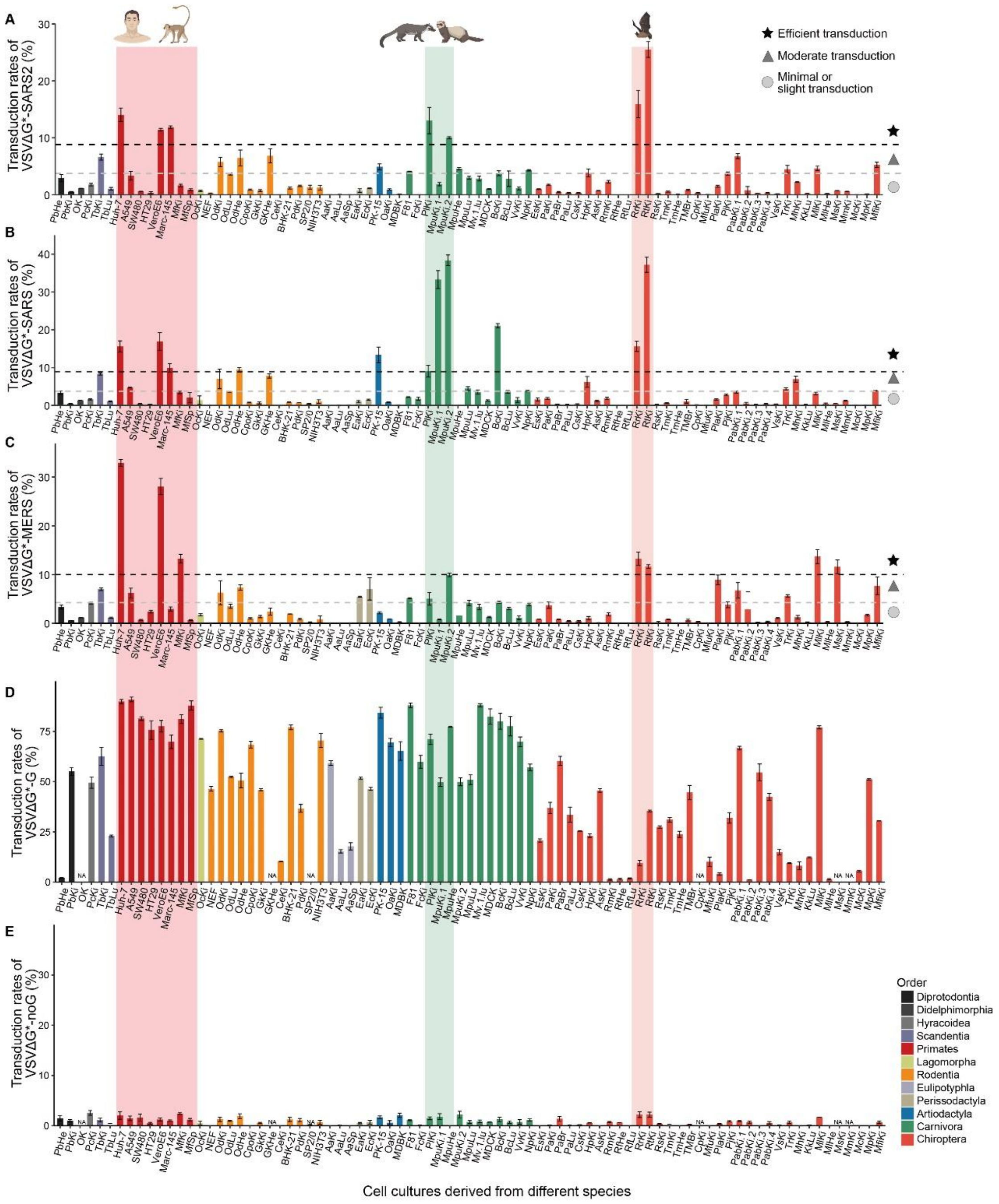

Fig. S3: VSVΔG*-SARS2, VSVΔG*-SARS, and VSVΔG*-MERS pseudotyped virus could efficiently transduce cell cultures from humans, non-human primates, carnivores, and bats. 83 cell cultures were in vitro infected by **(A)** VSVΔG*-SARS2, **(B)** VSVΔG*-SARS, **(C)** VSVΔG*-MERS, **(D)** VSVΔG*-G, and **(E)** VSVΔG*-noG pseudotyped viruses. The results highlight that apart from humans and non-human primates, several rodents (e.g., common degu), carnivores (i.e., ferret and palm civet), bats (like king horseshoe bat, Thomas horseshoe bat, and myotis laniger) should be monitored with higher priority. Cell cultures whose transduction rates

were lower than grey lines were classified as minimal- or slight- transduction; cell cultures whose transduction rates were above the black lines were classified as efficient transduction. The shadow highlights species whose cell cultures were most efficiently transduced by pseudotyped viruses, and should be monitored with prioritization. Three independent replicates were set for each infection assay. The bar represents Mean ± SD.

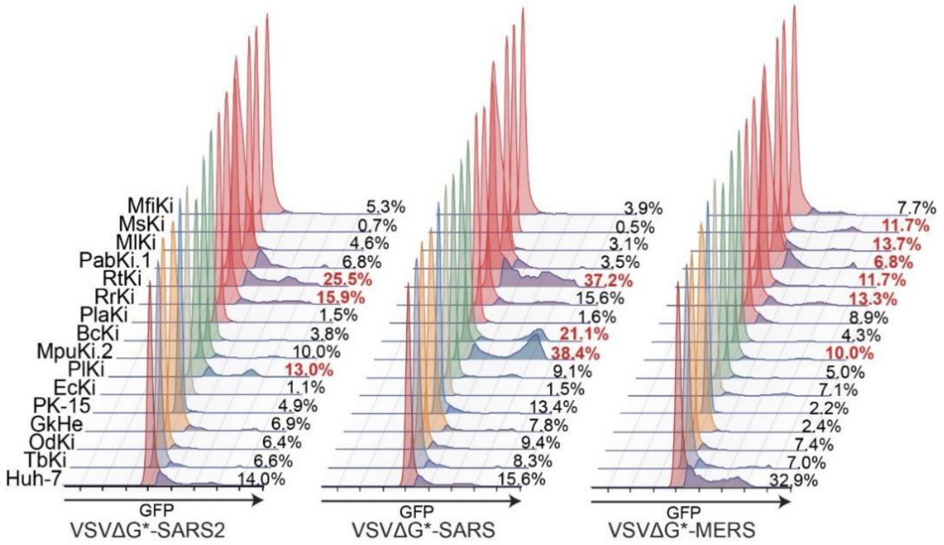

Fig. S4: Pseudotyped viruses showed different tropism when infect 83 cell cultures.

VSVΔG*-SARS displayed higher capacity than VSVΔG*-SARS2 to transduce ferret kidney MpuKi.2 cells, beagle dog kidney BcKi cells, and Thomas horseshoe bat kidney cells. While VSVΔG*-MERS showed higher transduction rates in ferret kidney MpuKi.2 cells and several bat cells. The colors of shadows represent the cell cultures were derived from different order (kindly see Figure 1). Three independent replicates were set for each infection assay.

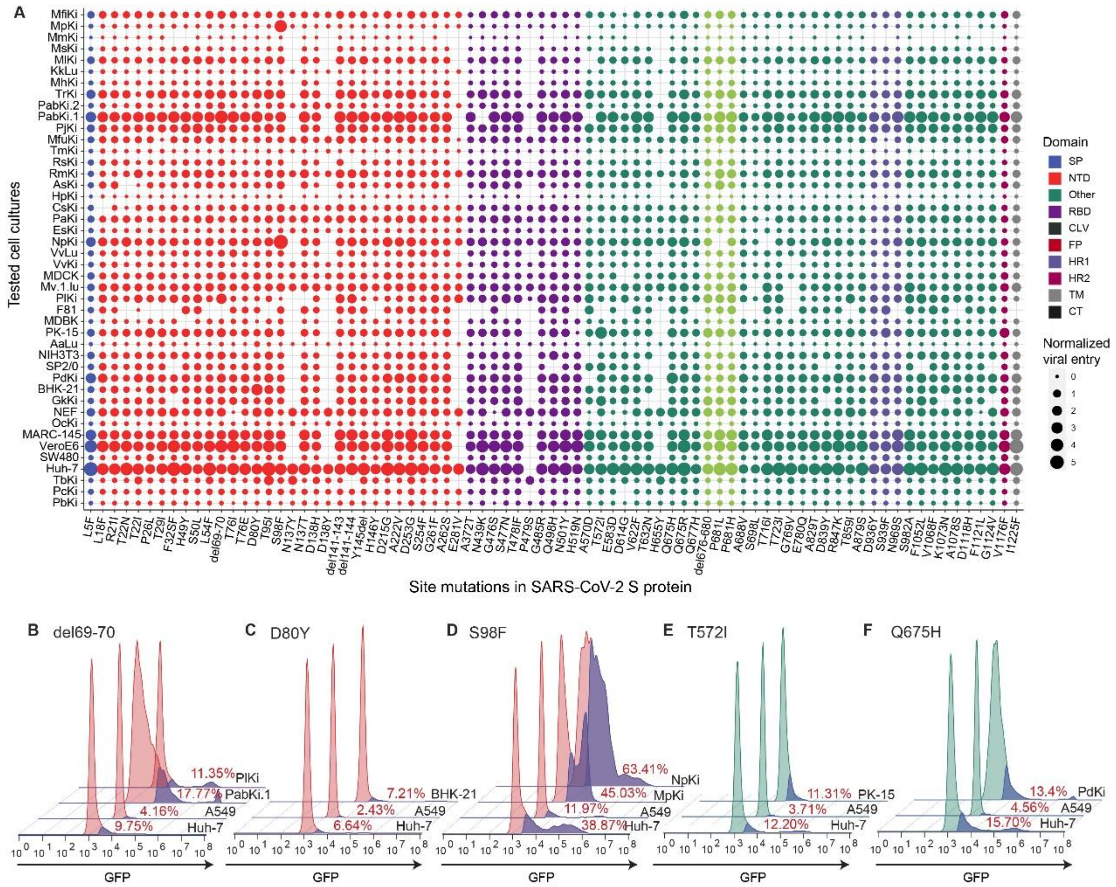

Fig. S5: Five mutations in S protein significantly altered the host tropism of VSVΔG*-SARS2. (A) A total of 79 pseudotyped viruses were generated with mutations from different domains of S proteins. The infection results of VSVΔG*-SARS2-Smut were displayed after being normalized to human lung adenocarcinoma (A549) cells. The color of the dot represents which domain it is located in, and the size of the dots presents the infection efficiencies compared to that of A549 cells. (B-F) Detail infection results of del69-70 (B), D80Y (C), S98F (D), T572I (E), and Q675H (F) towards certain cell cultures. Three independent replicates were set for each infection assay.

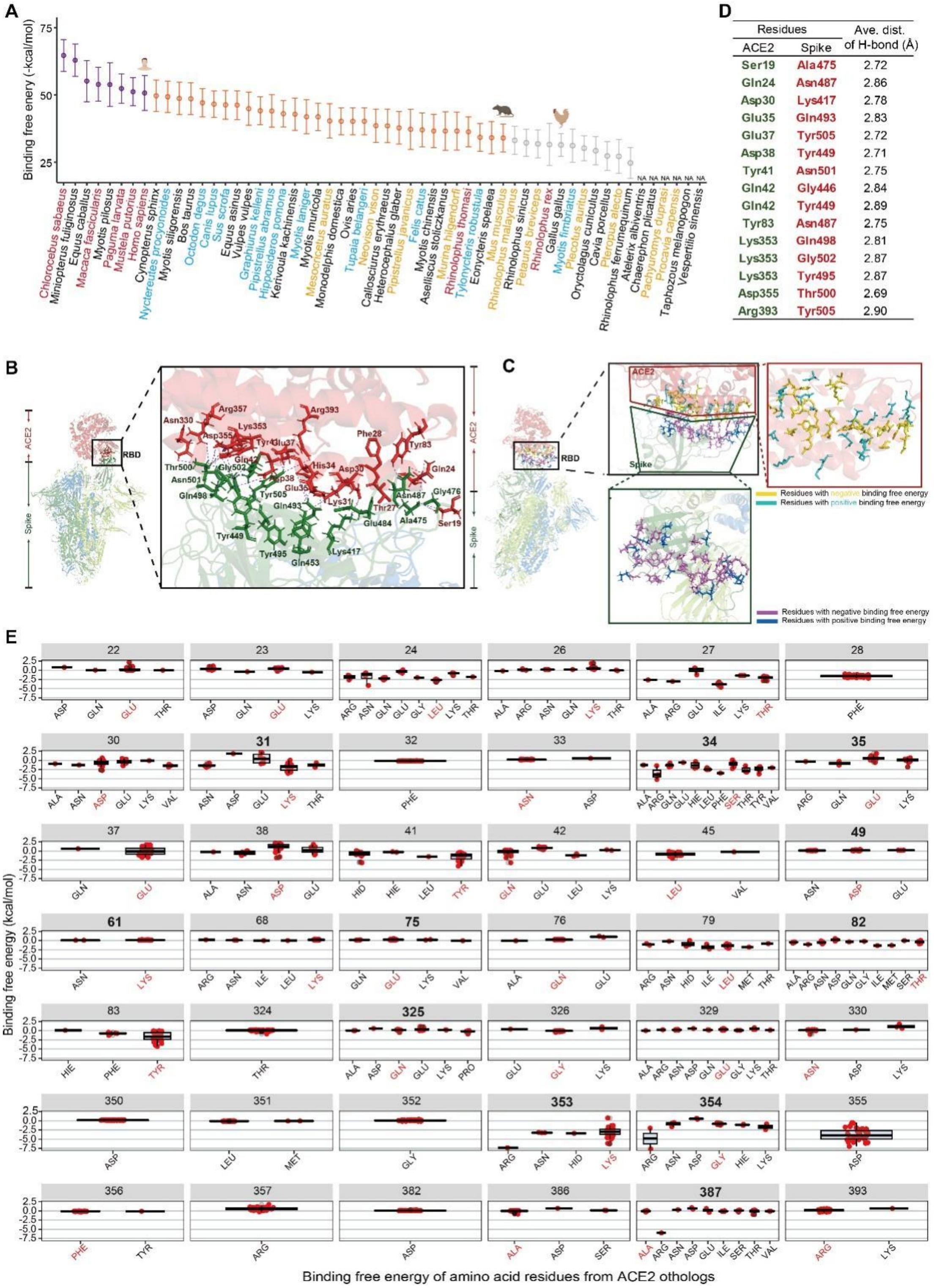

Residues		Ave. dist. of H-bond (Å)
ACE2	Spike	
Ser19	Ala475	2.72
Gln24	Asn487	2.86
Asp30	Lys417	2.78
Glu35	Gln493	2.83
Glu37	Tyr505	2.72
Asp38	Tyr449	2.71
Tyr41	Asn501	2.75
Gln42	Gly446	2.84
Gln42	Tyr449	2.89
Tyr83	Asn487	2.75
Lys353	Gln498	2.81
Lys353	Gly502	2.87
Lys353	Tyr495	2.87
Asp355	Thr500	2.69
Arg393	Tyr505	2.90

Fig. S6: Binding free energy contribution of different amino acid residues during the interaction of SARS-CoV-2 S-RBD and ACE2s. (A) The binding free energy between SARS-CoV-2 S-RBD and ACE2s from tested species. The binding free energy between SARS-CoV-2 S-RBD and chicken ACE2 was also calculated as negative control. The purple fonts represent species with binding free energy equal to

or higher than humans; the orange fonts represent species with binding free energy between mouse and human beings; the grey fonts represent species with binding free energy lower than mouse. **(B and C)** 3D models showed amino acid residues contribute to the binding of S-RBD and hACE2. **(D)** Key amino acid residues contribute to the interaction between S-RBD and hACE2. **(E)** Residues at 42 sites of ACE2 homologs contribute to the binding affinity of RBD-ACE2 complexes, with both positive and negative binding free energy. Negative binding free energy represents these residues contribute to the binding of corresponding complexes, while positive binding free energy represents these residues oppose the binding of corresponding complexes. These residues function together to provide a stable state of RBD-ACE2 complexes. The residues in red font represent the major type of amino residues at this site. The number with bold font represents these sites play important roles in cross-species transmission of SARS-CoV-2.

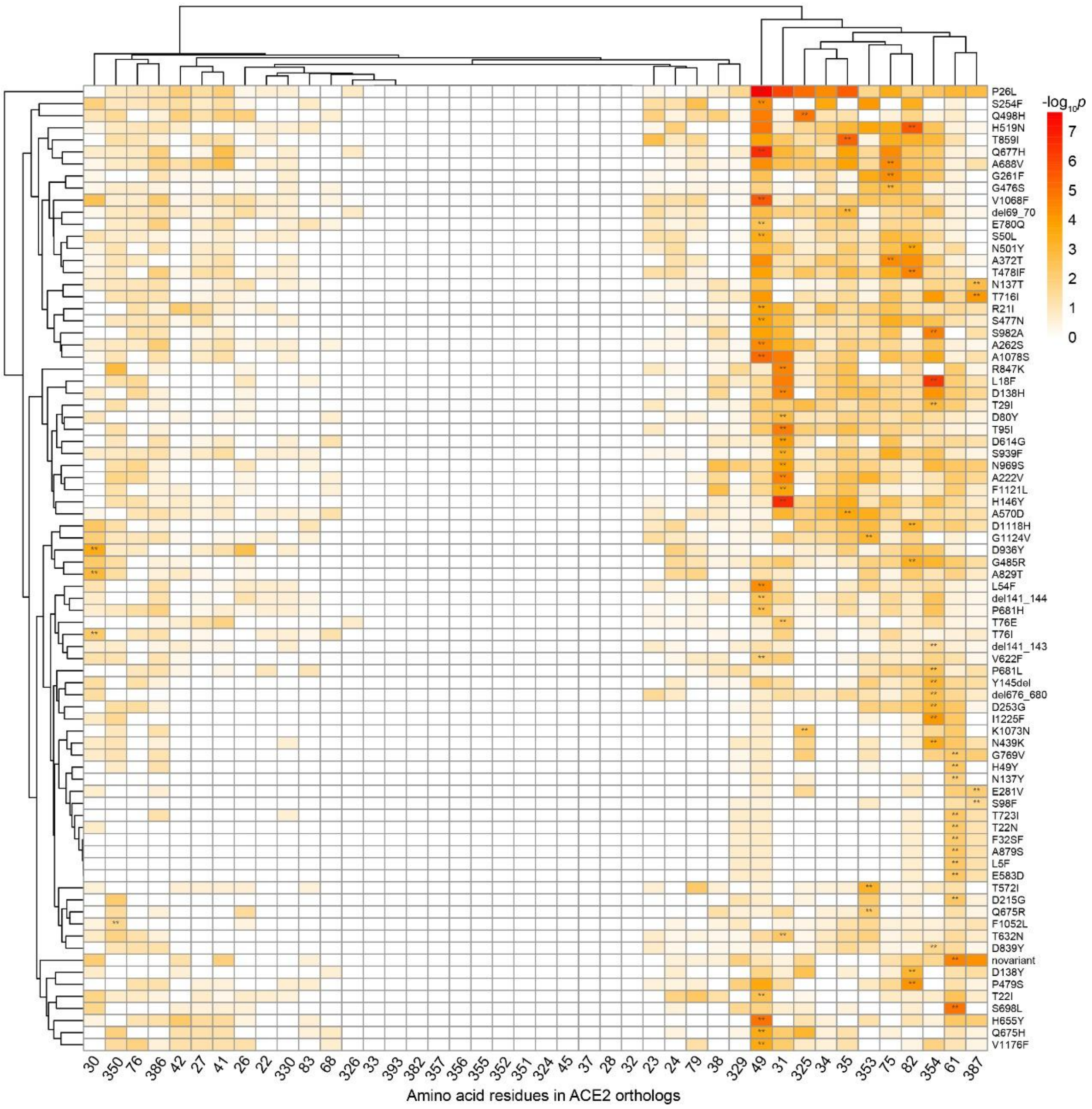

Fig. S7: Heatmap of association analyses of spike variants and ACE2 residues. The color indicates the *p* value estimated from Fisher's exact test of whether ACE2 residue (x axis) is evenly distributed in high and low infected groups determined by corresponding spike variants (y axis).

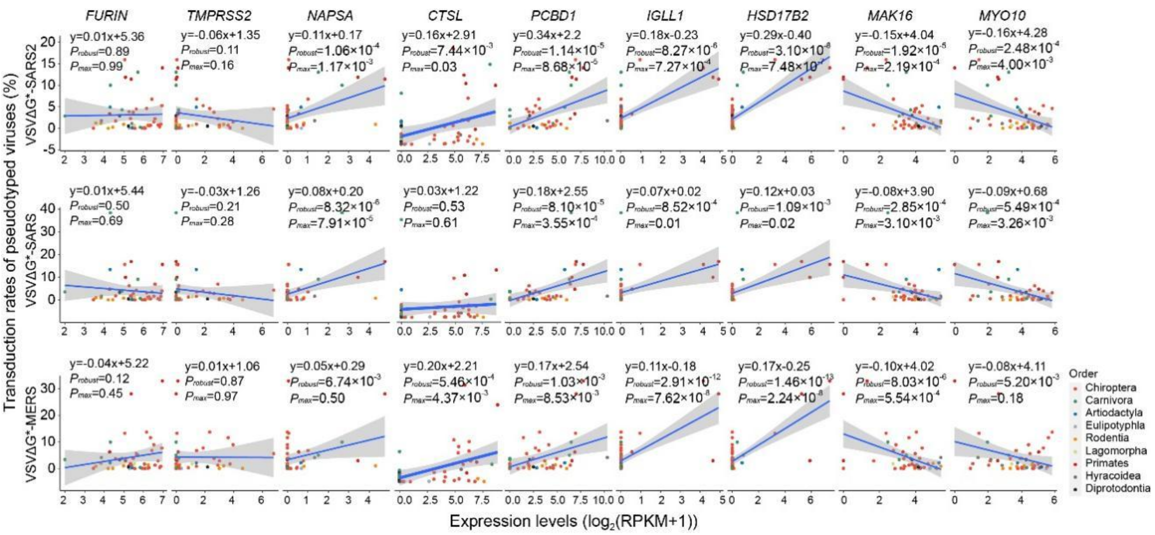

Fig S8: Liner regression of transduction rates and significantly enriched host factors.

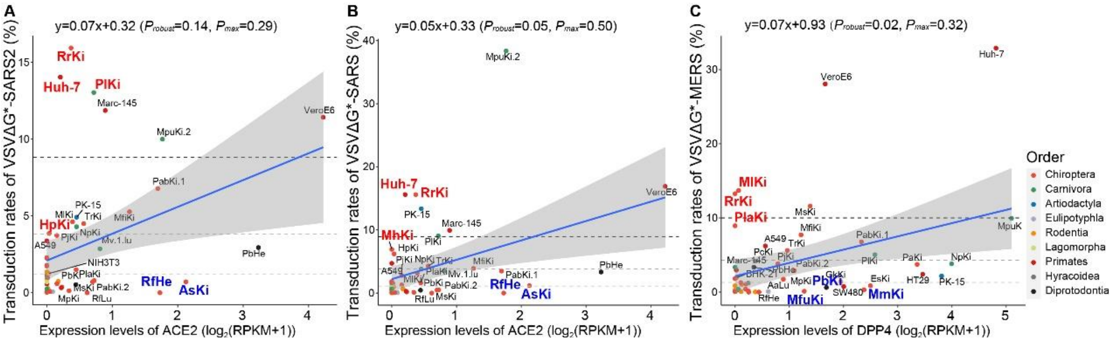

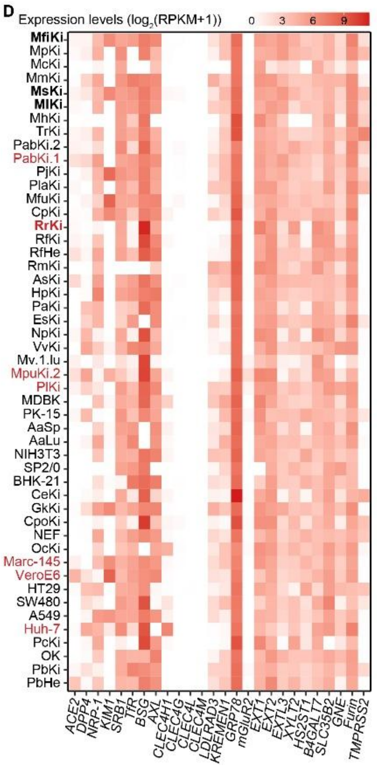

E

OLS regression of *ACE2s*, *furins*, *TMPRSS2s*, and other candidate recepotors or co-receptors to transduction rates of VSVΔG*-SARS2

Genes	Model Coefficient	Model Intercept	*P.value.all*	*P.value.robust*	*P.value.max*
ACE2	0.07412	0.31491	0.01573	0.13736	0.29280
DPP4	0.10027	0.94902	0.07615	0.02466	0.15118
NRP1	-0.08638	3.43866	0.15950	0.12568	0.27135
KIM1	0.13781	1.12492	0.18350	0.08421	0.29581
SRB1	0.04165	4.26547	0.43115	0.44087	0.80812
TfR	0.03428	0.05128	0.04448	0.65491	0.87790
BSG	0.20434	6.21873	0.04968	0.04551	0.13761
AXL	-0.05902	5.41808	0.35064	0.25271	0.80966
CLEC4H1	/	/	/	/	/
CLEC4G	-0.00440	0.05808	0.25665	0.39247	0.52694
CLEC4L	/	/	/	/	/
CLEC4M	/	/	/	/	/
LDLRAD3	-0.04650	1.79924	0.30923	0.31802	0.66266
KREMEN1	-0.04140	2.29955	0.42177	0.53452	0.93920
GRP78	-0.04492	8.30412	0.11524	0.16257	0.37241
mGluR2	0.02058	0.06935	0.08078	0.51980	0.61458
EXT1	0.03345	4.98867	0.34871	0.41585	0.94479
EXT2	-0.02206	5.11384	0.51703	0.18547	0.81083
EXTL3	-0.05896	3.24695	0.22195	0.16591	0.33581
XYLT2	0.02472	3.11084	0.46389	0.47195	0.57147
HS2ST1	-0.03321	2.72945	0.44777	0.28958	0.91743
B4GALT7	0.04637	3.82243	0.19448	0.20582	0.82244
SLC35B2	0.06777	4.30482	0.03805	0.00610	0.02272
GNE	-0.00627	2.63316	0.87672	0.95486	0.99598
Furin	0.00599	5.35657	0.87984	0.89307	0.99895
TMPRSS2	-0.06877	1.34633	0.24094	0.11384	0.16391

Fig. S9: Liner regression of transduction rates and relative receptors. (A and B) Expression levels of ACE2s displayed poor correlations with the transduction rates of VSVΔG*-SARS2 and VSVΔG*-SARS. **(C)** Expression levels of DPP4s displayed poor correlations with the transduction rates of VSVΔG*-MERS. The cell cultures in red font expressed low level of major receptors, but were moderately or efficiently transduced by pseudotyped viruses. While cell cultures in red font expressed low level of major receptors, but were minimally transduced by pseudotyped viruses. The light grey, grey, and black dash lines mean the definition of minimal-, slight-, moderate-, and efficient transduction by pseudotyped viruses. **(D)** Heatmap of expression levels of receptors, co-receptors, and catalytic enzymes. **(E)** Expression levels of receptors, co-receptors, and catalytic enzymes showed poor correlation with transduction rates of

VSVΔG*-SARS2. The cell cultures in red font were efficiently transduced cells by VSVΔG*-SARS2.

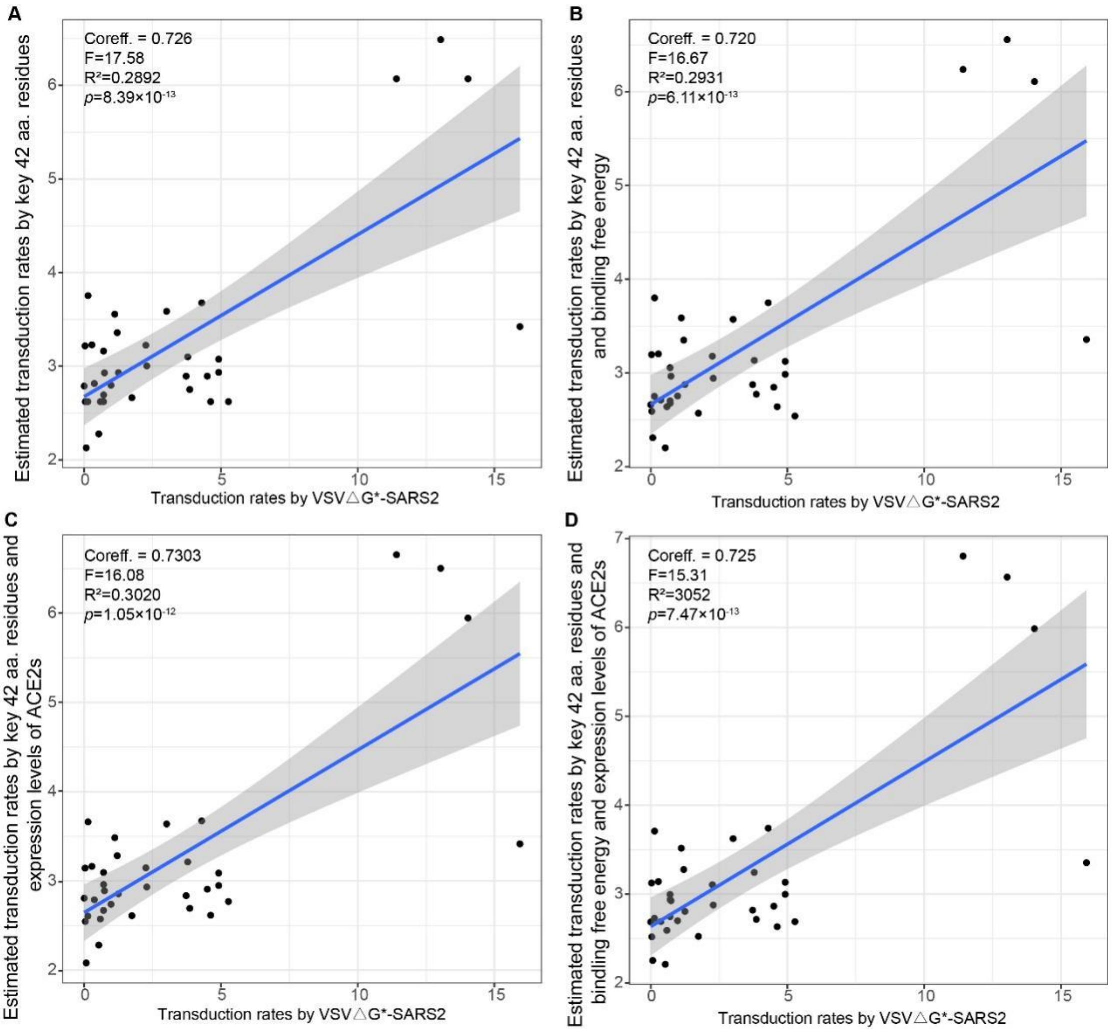

Fig. S10: The correlation between transduction rates and the estimated transduction rate using ACE2 sequence changes, free energy changes, and ACE2 expression.

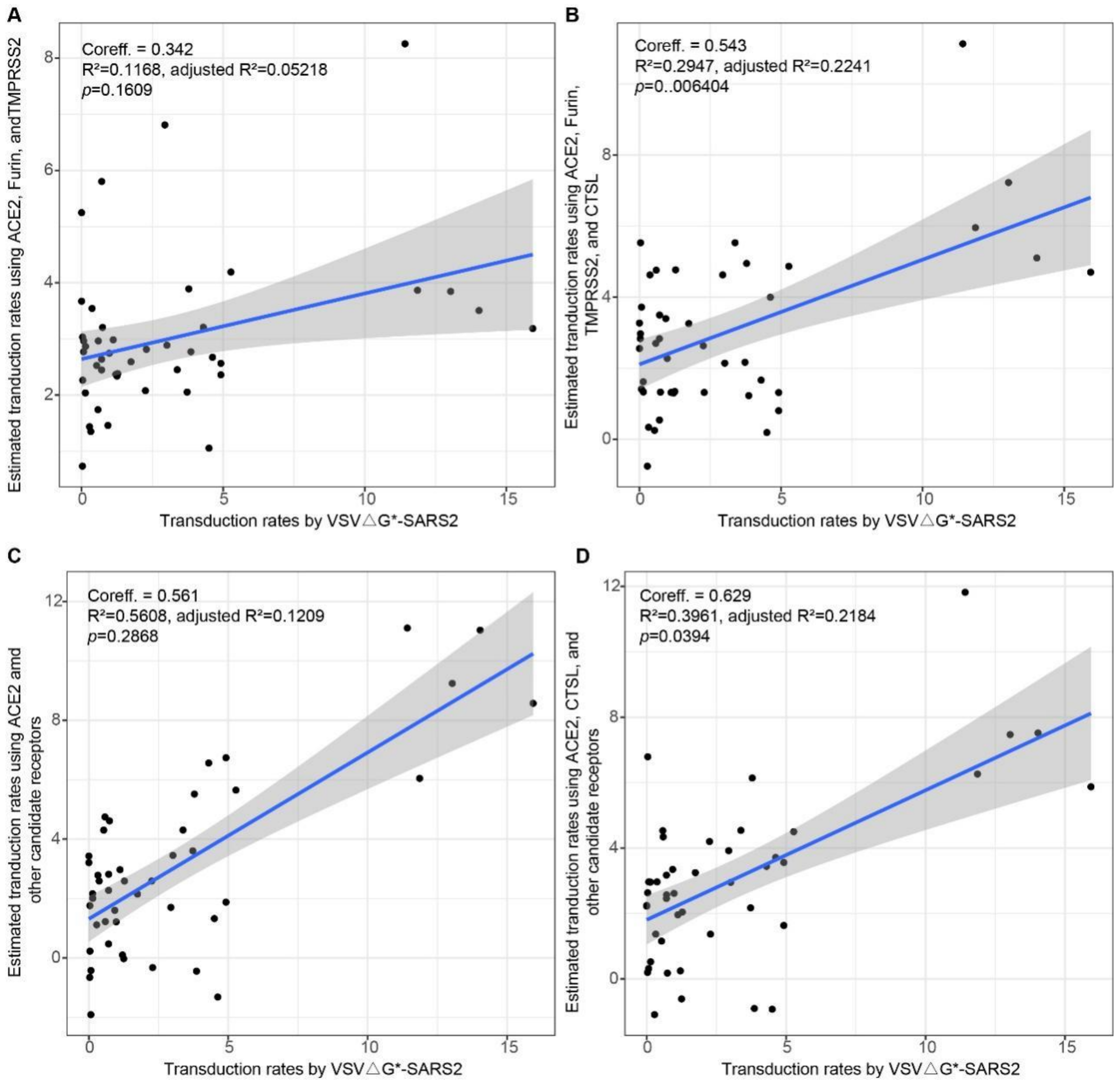

Fig. S11: The correlation between estimated transduction rates using expressions of ACE2, FURIN, and TMPRSS2 and transduction rates of VSVΔG*-SARS2.

Supplementary Tables：

Table S1: Transduction rates of cell cultures by SARS-CoV-2, SARS-CoV, MERS-CoV, VSV-G, and negative control pseudotyped viruses.

Table S2: Comparison between transduction results in this study with natural/experimental infection conclusions.

Table S3: Interactive analysis between variants in S protein and ACE2 orthologs with transduction rates of VSVΔG*-SARS2.

Table S4: List of 590 genes whose expression levels were significantly associated with transduction rates of VSVΔG*-SARS2.

Table S5: KEGG Pathway enrichment of 590 genes whose expression levels were significantly associated with transduction rate of VSVΔG*-SARS2.Over-represented KEGG terms were defined as having Fisher exact test $p \leqslant 0.05$.

Table S6: Biological Process (BP) GO term enrichment result of 590 genes whose expression levels were significantly associated with transduction rate of VSVΔG*-SARS2.Over-represented GO terms were defined as having Fisher exact test $p \leqslant 0.05$.

Table S7: List of 453 genes whose expression levels were significantly associated with the transduction rates of VSVΔG*-SARS.

Table S8: KEGG Pathway enrichment of 453 genes whose expression levels were significantly associated with transduction rate of VSVΔG*-SARS. Over-represented KEGG terms were defined as having Fisher exact test $p \leqslant 0.05$.

Table S9: Biological Process (BP) GO term enrichment result of 453 genes whose expression levels were significantly associated with transduction rate of VSVΔG*-SARS. Over-represented GO terms were defined as having Fisher exact test $p \leqslant 0.05$.

Table S10: List of 416 genes whose expression levels were significantly associated with the transduction rates of VSVΔG*-MERS.

Table S11: KEGG Pathway enrichment of 416 genes whose expression levels were significantly associated with transduction rate of VSVΔG*-MERS. Over-represented KEGG terms were defined as having Fisher exact test $p \leqslant 0.05$.

Table S12: Biological Process (BP) GO term enrichment result of 416 genes whose expression

levels were significantly associated with transduction rate of VSVΔG*-MERS.Over-represented GO terms were defined as having Fisher exact test $p \leqslant 0.05$.

Table S13: List of 95 overlapped genes whose expression levels were significantly associated with the transduction rates of VSVΔG*-SARS2, VSVΔG*-SARS, and VSVΔG*-MERS.

Table S14: Sample information used in this project.

Table S15: Mapping rates of different RNA-seq samples to reference genome.

Table S16: List and gene length of ortholog genes.

Table S17: The Normalized expression (log2(RPKM+1)) and raw expression levels of different transcripts in tested cell cultures. Most of the samples have two replicates, while a few samples (i.e., CpoKi, AsSp, McKi, PlKi, RmKi, etc.) have one replicate either because of low RNA quality or other factors such as insufficient coverage or low mapping rate.
